# Volumetric Absorptive Microsampling (VAMS) for Therapeutic Drug Monitoring of Antiseizure Medications (ASMs) in Pediatric Patients

**DOI:** 10.3390/ph19081188

**Published:** 2026-07-29

**Authors:** Raffaele Simeoli, Alessandro Mancini, Sara Cairoli, Chiara Rossi, Costanza Calabrese, Marina Trivisano, Licia Salimbene, Carlo Dionisi Vici, Nicola Pietrafusa, Nicola Specchio, Bianca Maria Goffredo

**Affiliations:** 1 Division of Metabolic Diseases and Hepatology, Bambino Gesù Children’s Hospital, IRCCS, 00146 Rome, Italy; raffaele.simeoli@opbg.net (R.S.); alessandro.mancini@opbg.net (A.M.); sara.cairoli@opbg.net (S.C.); chiara.rossi@opbg.net (C.R.); carlo.dionisivici@opbg.net (C.D.V.); 2 Neurology, Epilepsy, and Movement Disorders Unit, Full Member of European Reference Network EpiCARE, Bambino Gesù Children’s Hospital, IRCCS, 00146 Rome, Italy; costanza.calabrese@opbg.net (C.C.); marina.trivisano@opbg.net (M.T.); licia.salimbene@opbg.net (L.S.); nicola1.pietrafusa@opbg.net (N.P.); nicola.specchio@opbg.net (N.S.)

**Keywords:** volumetric absorptive microsampling (VAMS), therapeutic drug monitoring (TDM), LC–MS/MS, bioanalytical method validation, antiseizure medications, pediatric patients, microsampling, precision dosing

## Abstract

**Background:** Volumetric absorptive microsampling (VAMS) is an emerging tool for therapeutic drug monitoring (TDM) of several drugs including antiseizure medications (ASMs). Here, we compared the concentrations of carbamazepine (CBZ), levetiracetam (LEV), lacosamide (LCS), topiramate (TPR) and the benzodiazepine (BZ) clobazam (CLB) in plasma and VAMS samples. **Methods**: VAMS samples were collected by fingerprick in pediatric patients followed at our center. Patients were also subjected to conventional venous blood sampling. Plasma and VAMS samples were analyzed by using a UHPLC-MS/MS validated kit for AEs and BZs (ClinMass LC-MS/MS Complete Kit^®^). A cross-validation analysis was performed by using Spearman correlation (rho), Deming regression and Bland–Altman plots. **Results:** Two analytical methods for measuring selected AEs and BZs in VAMS samples were developed and validated in accordance with the ICH M10 guidelines. Based on Bland–Altman results, a satisfactory agreement was observed between VAMS and plasma for CBZ, LCS, TPR, LEV and N-CLB. Considering the absence of interchangeability between capillary blood and plasma levels for CBZ-Diol, -Epoxi and CLB, a blood to plasma ratio was used to convert VAMS values into estimated plasma concentrations. Comparison of estimated vs. observed plasma results showed a successful predictive performance for this conversion approach. **Conclusions:** A positive agreement between plasma and VAMS was found for CBZ, LCS, TPR, LEV and N-CLB. Conversely, a conversion factor based on blood to plasma ratio should be adopted to convert CBZ-Diol, -Epoxi and CLB VAMS results into estimated plasma concentrations. This study confirmed the utility of VAMS for TDM of selected ASMs in pediatric patients during routine clinical practice.

## 1. Introduction

Epilepsy is a serious neurological condition characterized by a cumulative annual incidence of 61.44 per 100,000 person-years and a significant impact on both everyday life and health system costs [1]. Pharmacological treatments are symptomatic and provide an immediate control of seizures with the ultimate aim to prevent both medium and long-term seizure relapse [2]. However, it has been estimated that almost 30% of patients are affected by drug-resistant epilepsy, highlighting the need for alternative and innovative medications.

Antiseizure treatments are often based on a combination of different medications including both AEs and BZs rather than a monotherapy, thereby increasing the chance of pharmacokinetic (PK) and pharmacodynamic (PD) drug–drug interactions (DDIs) [3]. However, both old and new-generation ASMs are characterized by a non-linear PK that makes plasma concentration poorly predictable, thereby exposing patients to different adverse events mainly involving the central nervous system (CNS) and the cognitive area. As consequence, physiological processes that differentiate children from adults in terms of organ maturation and developmental changes represent an additional source of variability in pediatric patients compared to adults and are responsible for both the response to treatments and the occurrence of side effects in these subjects.

Therefore, therapeutic drug monitoring (TDM) has become a valuable tool for the optimization of pharmacological treatments in epilepsy [4]. In fact, there are several conditions in which TDM of ASMs is strongly recommended. These include the occurrence of adverse drug reactions (ADRs), the need for dose adjustment in special populations (e.g., children, elderly, patients with associated diseases, drug formulation changes, pregnancy), or when an interacting drug is added or removed. Similarly, the assessment of compliance and the optimization of individual therapeutic concentrations represent additional scenarios where TDM is particularly indicated [4].

Notably, physiological and ethical concerns are often a limit to the application of TDM in neonatal and pediatric populations mainly due to the invasiveness of blood sampling by conventional venipuncture [5]. In order to overcome this limitation, several microsampling alternatives have been proposed [6]. Dried blood spot (DBS) is widely used in neonatal screening programs and for qualitative analyses, although there is increasing interest in the use of DBS for quantitative applications, including TDM [6,7]. However, mainly due to the hematocrit (hct) effect that influences this sampling procedure, the use of DBS for quantitative measurements introduces additional analytical questions. In fact, appropriate validation is required by comparing unknown concentrations with those measured in conventionally collected blood or plasma samples [7]. Alternatively, the heel stick capillary (HSC) device represents a valid strategy to collect small blood samples in neonates and to perform therapeutic drug monitoring of several drug classes including antifungals [8].

In recent years, volumetric absorptive microsampling (VAMS) has been proposed as a feasible alternative to DBS for quantitative applications. Similar to DBS, this method is minimally invasive and virtually painless, an aspect that makes it particularly suitable for pediatric patients. However, compared to DBS, previous evidence has reported that VAMS is not affected by the HCT value [6,9]. These devices consist of a plastic support with a hydrophilic polymer tip, designed to collect fixed volumes of blood by capillarity (10, 20, or 30 μL) according to the tip size. Once collected, VAMS samples are left to dry and stored until the analysis [10].

So far, VAMS has been tested for different TDM applications, by comparing the obtained drug’s concentrations with those measured in plasma or DBS samples [11,12,13,14,15,16,17]. Recently, we have proposed the use of VAMS and dried plasma spot (DPS) for TDM of antifungal triazole agents in pediatric patients [18]. Similarly, these microsampling devices have been used for monitoring blood concentrations of both old and new-generation ASMs, including cannabidiol [19,20,21,22,23]. However, although the studies published so far have shown VAMS validation in “real-life” clinical samples, in only few of them was sampling performed in adult and pediatric patients by collecting capillary blood through fingerprick [22,23,24]. In particular, in the study conducted on adult patients with epilepsy (PWEs) by Cancellerini C. and colleagues (2024), the authors have compared plasma concentrations of different antiepileptic drugs (AEDs) with those measured in VAMS samples autonomously collected by patients (self-collected VAMS) and by nurses (nurse-collected VAMS) [24].

Here, we have developed and validated two LC-MS/MS methods for the quantification of four AEs and one benzodiazepine (BZ) in VAMS samples. Specifically, following the ASMs were analyzed: carbamazepine (CBZ) with its metabolites diol (CBZ-Diol) and epoxide (CBZ-Epoxi), levetiracetam (LEV), lacosamide (LCS), topiramate (TPR) and the benzodiazepine (BZ) clobazam (CLB) with its active metabolite N-desmethylclobazam (or norclobazam, N-CLB). Thereafter, clinical applicability of the validated methods was tested in pediatric patients affected by epilepsy and followed at our center. In particular, drug concentrations found in plasma samples conventionally obtained by venipuncture were compared to those detected in nurse-collected VAMS by fingerprick. The aim of this study was to validate the use of VAMS for monitoring blood levels of the selected drugs during routine clinical practice and to promote the use of remote TDM in a pediatric outpatient setting.

## 2. Results

Table 1 summarizes hallmarks, key data and main findings of our comparative analytical study.

### 2.1. Calibration Curve and Linearity Evaluation

Six-point calibration curves used for measuring drug concentrations in VAMS samples are displayed in Supplementary Figure S1A–H (blue triangles indicate L-QC, M-QC, and H-QC samples). For each analyte, calibration curves were linear over the ranges reported in Supplementary Table S1. Similarly, regression coefficients (R^2^) were close to 1 for the analyzed compounds (Supplementary Table S1).

Linearity was evaluated on five different calibration curves. Each calibrator was measured within 15% of the nominal concentration. To further evaluate the linearity of each calibration curve, back-calculated concentrations for the calibration standards were evaluated, and accuracy (expressed as %bias) was also calculated by comparing predicted to nominal concentrations. For each calibration standard, the %bias was within the acceptable value of ≤15%.

### 2.2. Selectivity and Specificity

**Figure 1:**
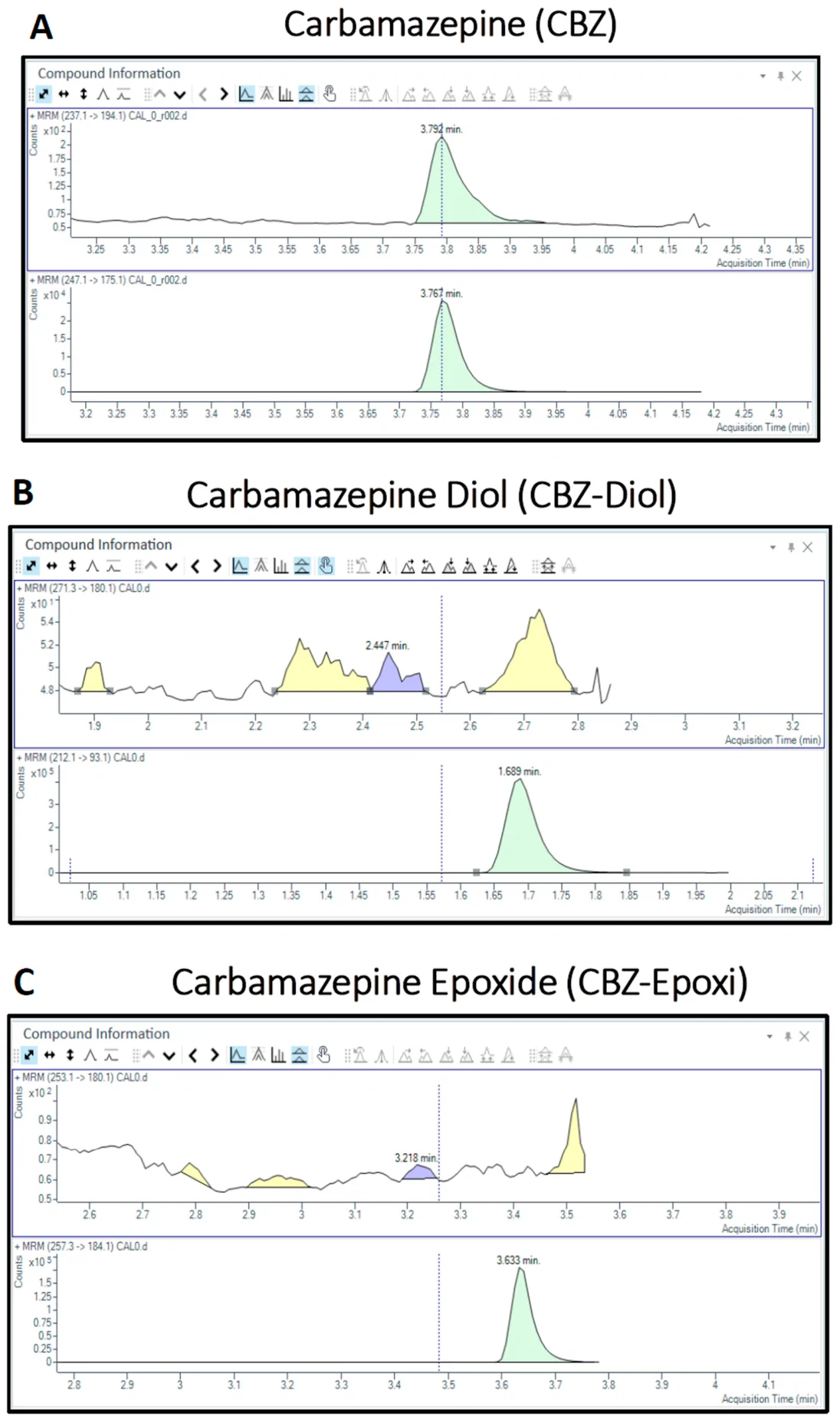

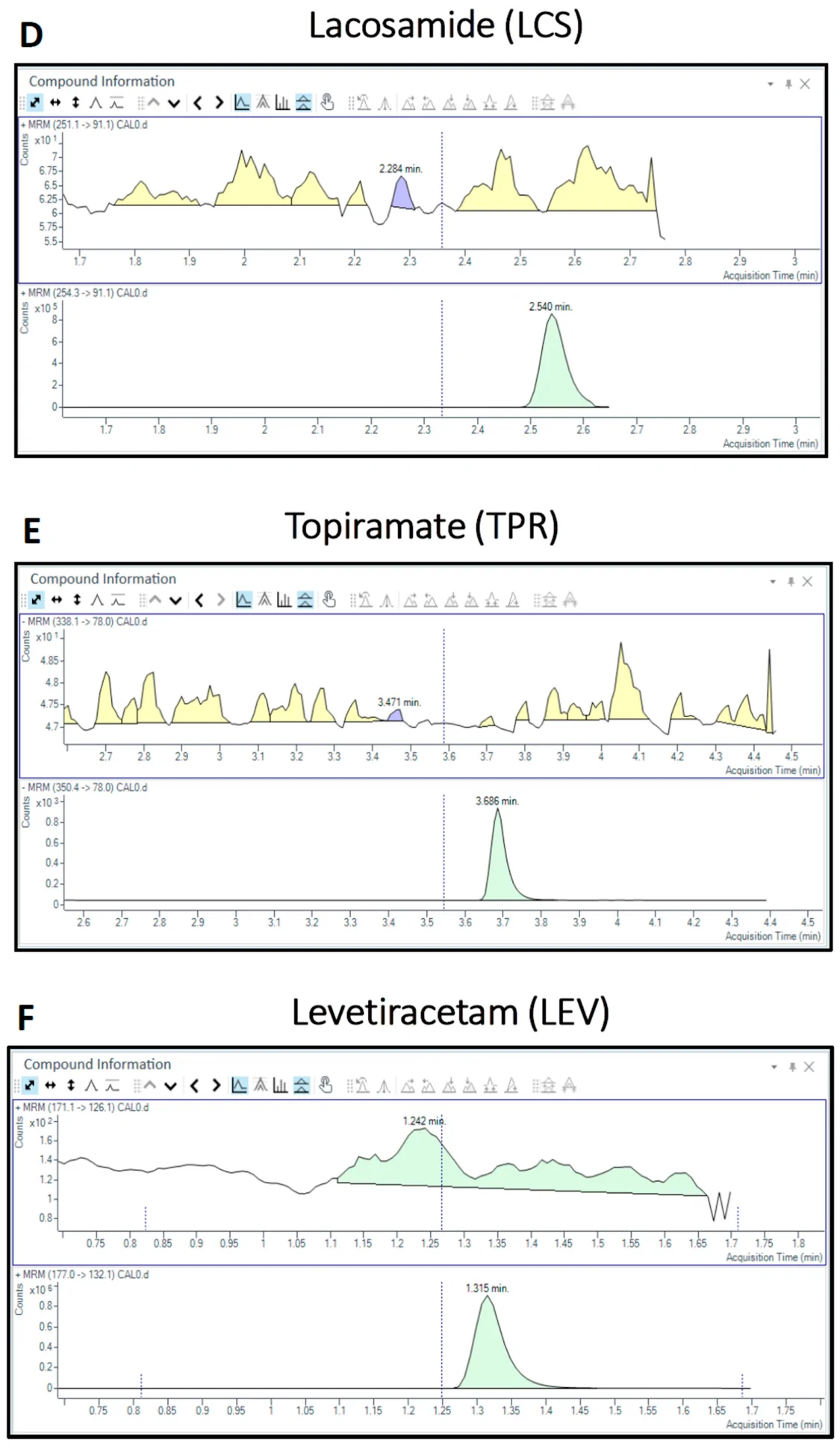

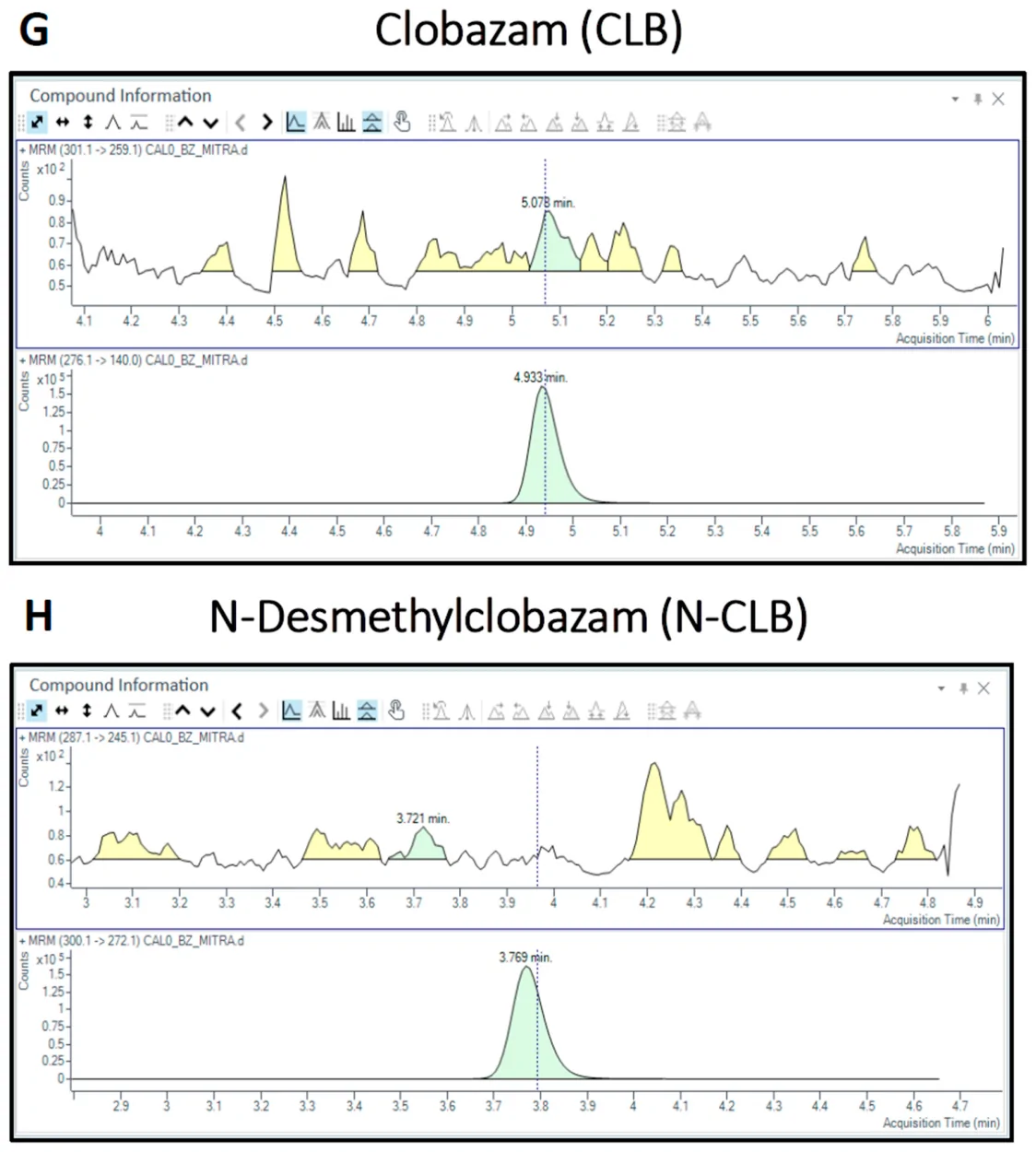
Chromatograms of VAMS blank sample spiked with deuterated IS for carbamazepine (**A**), carbamazepine-diol (**B**), carbamazepine-epoxide (**C**), lacosamide (**D**), topiramate (**E**), levetiracetam (**F**), clobazam (**G**) and N-desmethylclobazam (**H**). For each chromatogram, the upper and lower layers indicate the fragments used as the quantifier and the internal standard compound, respectively. The relative response (counts) from the baseline and the acquisition time (min) are reported on the y- and x-axes, respectively. For each peak, the retention time is displayed.

Six different VAMS samples were prepared from drug-free whole blood and samples spiked with the internal standard (IS) mix in order to evaluate the presence of possible interferences with the target analyte detection. As reported in Figure 1A–H, blank samples spiked with IS did not show interfering peaks within the displayed chromatograms. Moreover, the median signal of these blank samples was below 20% of the LLOQ, thereby ensuring the selectivity of the method.

**Figure 2:**
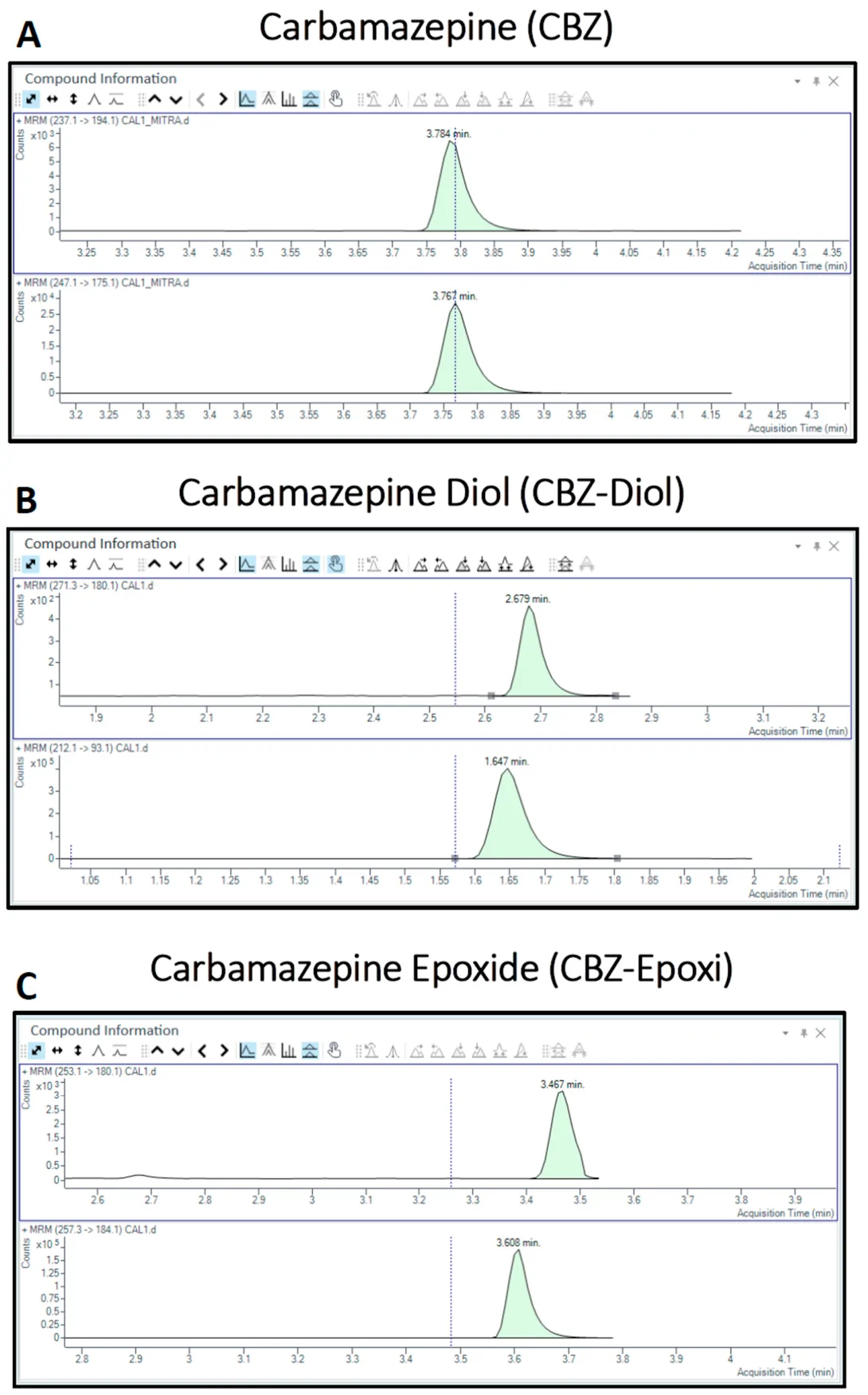

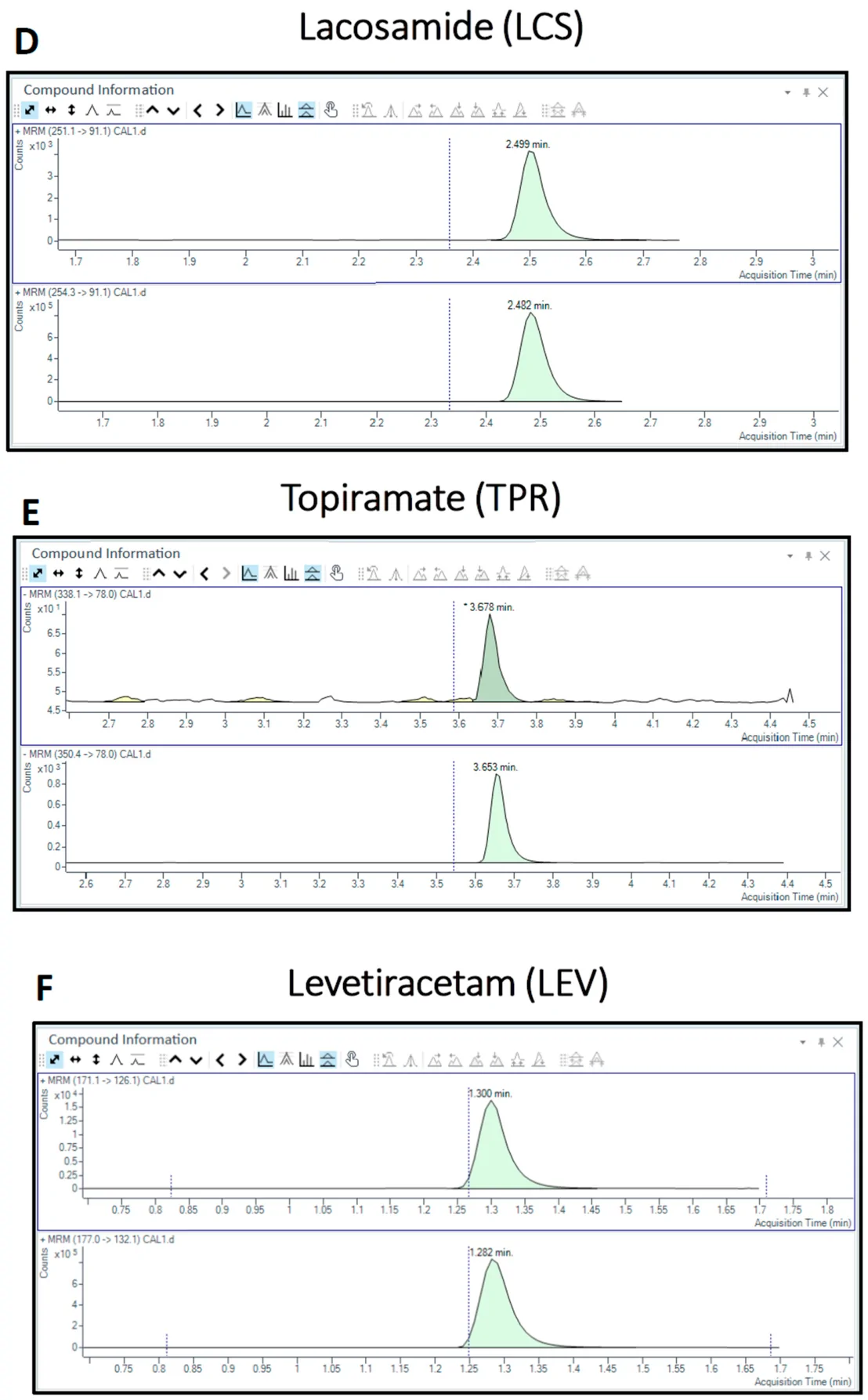

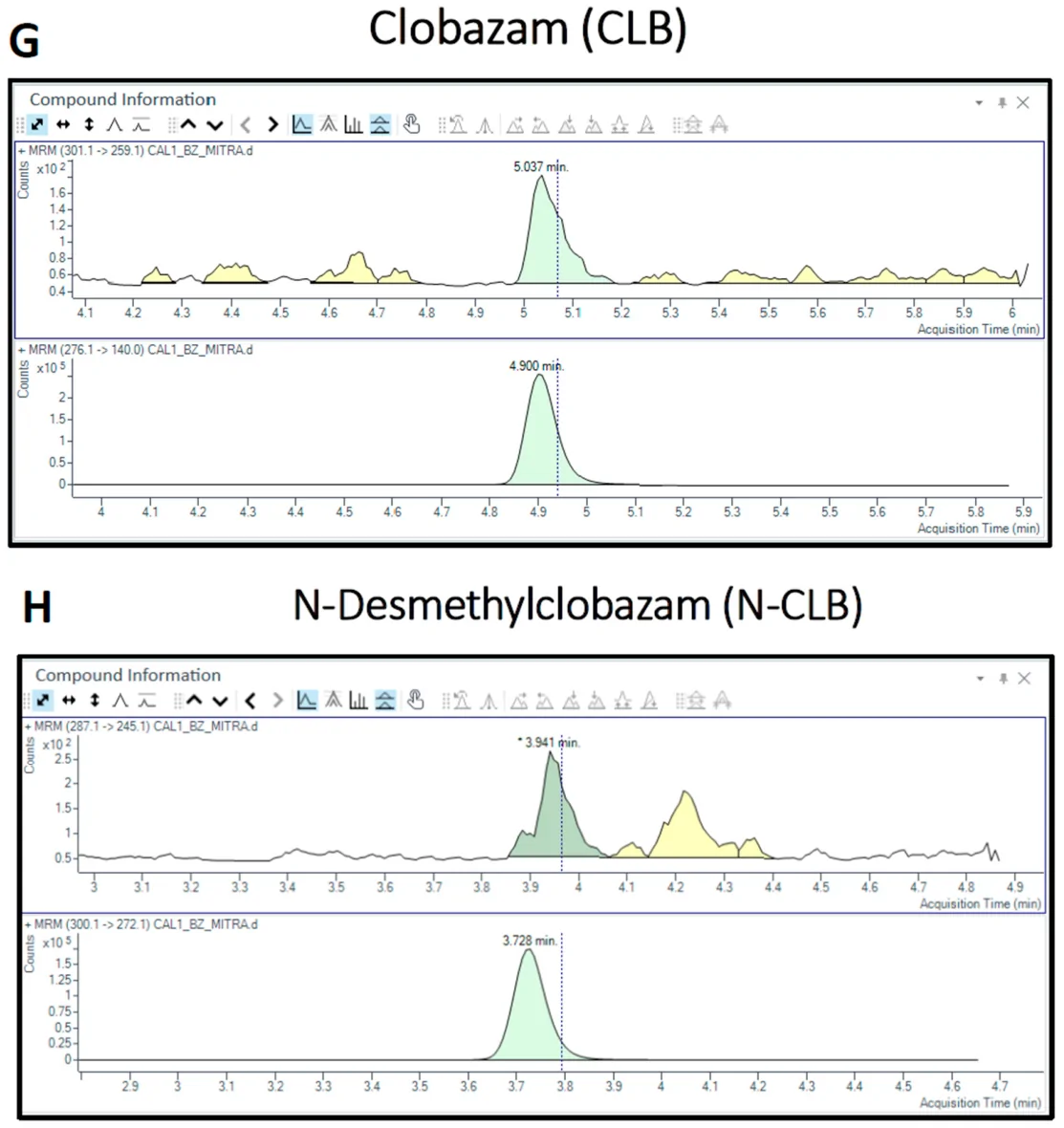
Chromatograms of VAMS LLOQ for carbamazepine (**A**), carbamazepine-diol (**B**), carbamazepine-epoxide (**C**), lacosamide (**D**), topiramate (**E**), levetiracetam (**F**), clobazam (**G**) and N-desmethylclobazam (**H**). For each chromatogram, the upper and lower layers indicate the fragments used as the quantifier and the internal standard compound, respectively. The relative response (counts) from the baseline and the acquisition time (min) are reported on the y- and x-axes, respectively. For each peak, the retention time is displayed. * denote the quantifier fragment peaks employed for quantitative analysis.

LLOQ chromatograms for each tested compound are displayed in Figure 2A–H. Moreover, both intra- and inter-assay accuracy and precision evaluated at the LLOQ level were within 20% for the selected analytes. Results are reported in Table 2 and Table 3.

Finally, in order to assess the presence of carry-over, blank VAMS samples were prepared and spiked with IS. These samples were run in triplicate after the highest calibration point for each target analyte. According to the ICH M10 guideline on bioanalytical method validation and study sample analysis, the median signal of these VAMS blank samples was less than 20% of the LLOQ and 5% of the IS, confirming the absence of carry-over.

### 2.3. Accuracy and Precision

Intra- and inter-assay accuracy and precision were assessed at LLOQ, L-QC, M-QC and H-QC levels (Table 2 and Table 3). Specifically, the intra- and inter-assay accuracy (expressed as mean %bias) and precision (reported as %CV) were ≤15% for each QC level and ≤20% for the LLOQ, in accordance with the ICH M10 guideline on bioanalytical method validation and study sample analysis.

The matrix effect (ME) was also evaluated on *n* = 3 VAMS samples prepared at low and high QC levels for each tested compound. As reported in Table 4, ME% resulted in values within the acceptable range (85–115%) for the selected analytes. Similarly, results of extraction recovery (ER%) ranged from 94 to 102% for the analyzed VAMS samples. Results are displayed in Table 4.

### 2.4. Stability Evaluation

Stability was evaluated on VAMS samples prepared at low, medium and high levels and stored at room temperature in the appropriate cassette for up to 28 days. The % difference was calculated by comparing the drugs’ concentrations measured at each time point to that obtained on the preparation day (Time 0). After 7 days, the % difference ranged from 0.14 to 12.19% at medium and high QC levels for CBZ-Diol, CBZ-Epoxi, LCS, LEV and TPR. Conversely, 7 days after sample preparation, the % difference at low QC levels ranged from 2.70% for LEV to 84.1% for CBZ-Diol. After 14 and 28 days, the % differences showed an increasing trend at low, medium and high QC levels for each analyzed AE drug. The only exception is represented by CBZ that showed higher % differences at every QC level 7 days after initial VAMS preparation (Time 0) (Table 5).

After 7 days, the medium and high QCs of CLB and N-CLB showed a % difference range of 8.91–8.94% and 14.75–21.02%, respectively. Similarly, the % difference for CLB and N-CLB at medium and high QC levels ranged from 10.16 to 31.31% after 14 days and from 20.82 to 42.44% after 28 days. In contrast, stability of CLB and N-CLB after 7 days at a low QC level showed a % difference of 16.98 and 8.62, respectively. However, these percentages increased after 14 and 28 days at room temperature. In fact, the % difference for CLB and N-CLB at a low QC level ranged from 32.54 to 85.60% after 14 days and from 46.53 to 93.08% after 28 days (Table 5).

We have also evaluated the stability of extracted low-, medium- and high-QC samples following two cycles of freezing (−20 °C) and thawing over a 1-month period. Following a first cycle, the % difference ranged from 0.27 to 9.28% for the tested analytes. Similar percentages were obtained after a second freeze–thaw cycle (0.1–12.18%) performed 15 days after the previous one (Table 6).

### 2.5. Comparison Between Sampling Methods: Capillary Whole Blood VAMS vs. Plasma Samples Collected by Venipuncture

**Figure 3:**
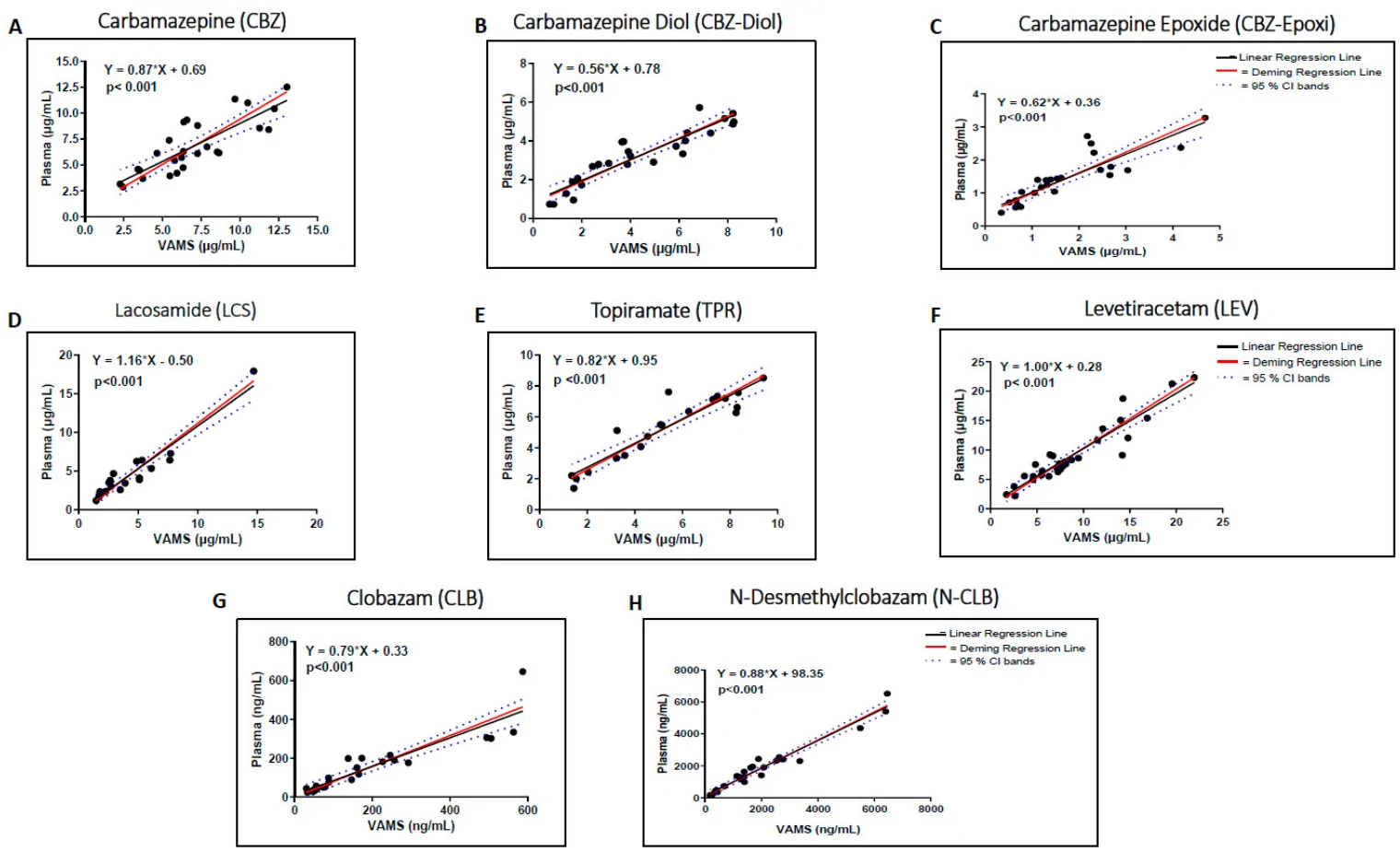
Passing–Bablok correlation plots. For VAMS samples, Passing–Bablok fits were: y = 0.87 × X + 0.69 for carbamazepine (**A**), y = 0.56 × X + 0.78 for carbamazepine-diol (**B**), y = 0.62 × X + 0.36 for carbamazepine-epoxide (**C**), y = 1.16 × X − 0.50 for lacosamide (**D**), y = 0.82 × X + 0.95 for topiramate (**E**), y = 1.00 × X + 0.28 for levetiracetam (**F**), y = 0.79 × X + 0.33 for clobazam (**G**), y = 0.88 × X + 98.35 for N-Desmethylclobazam (**H**).

Passing–Bablok correlation plots for the selected ASMs are shown in Figure 3. Moreover, both slope and intercept values are reported in Table 7 with relative 95% confidence interval (CI). As reported, a constant bias was found exclusively for CBZ-Diol, -Epoxi and TPR since the 95% CI intercept did not include 0. For the same analytes, a proportional bias was also observed since the 95% CI slopes did not include 1 (Table 7).

The Spearman’s correlation coefficient (rho) ranged from 0.83 to 0.95 for the selected compounds, indicating a positive correlation between drug concentrations measured in plasma and VAMS samples (Table 7). Moreover, significant *p* values (*p* < 0.001) were also observed for the described correlations (Figure 3).

**Figure 4:**
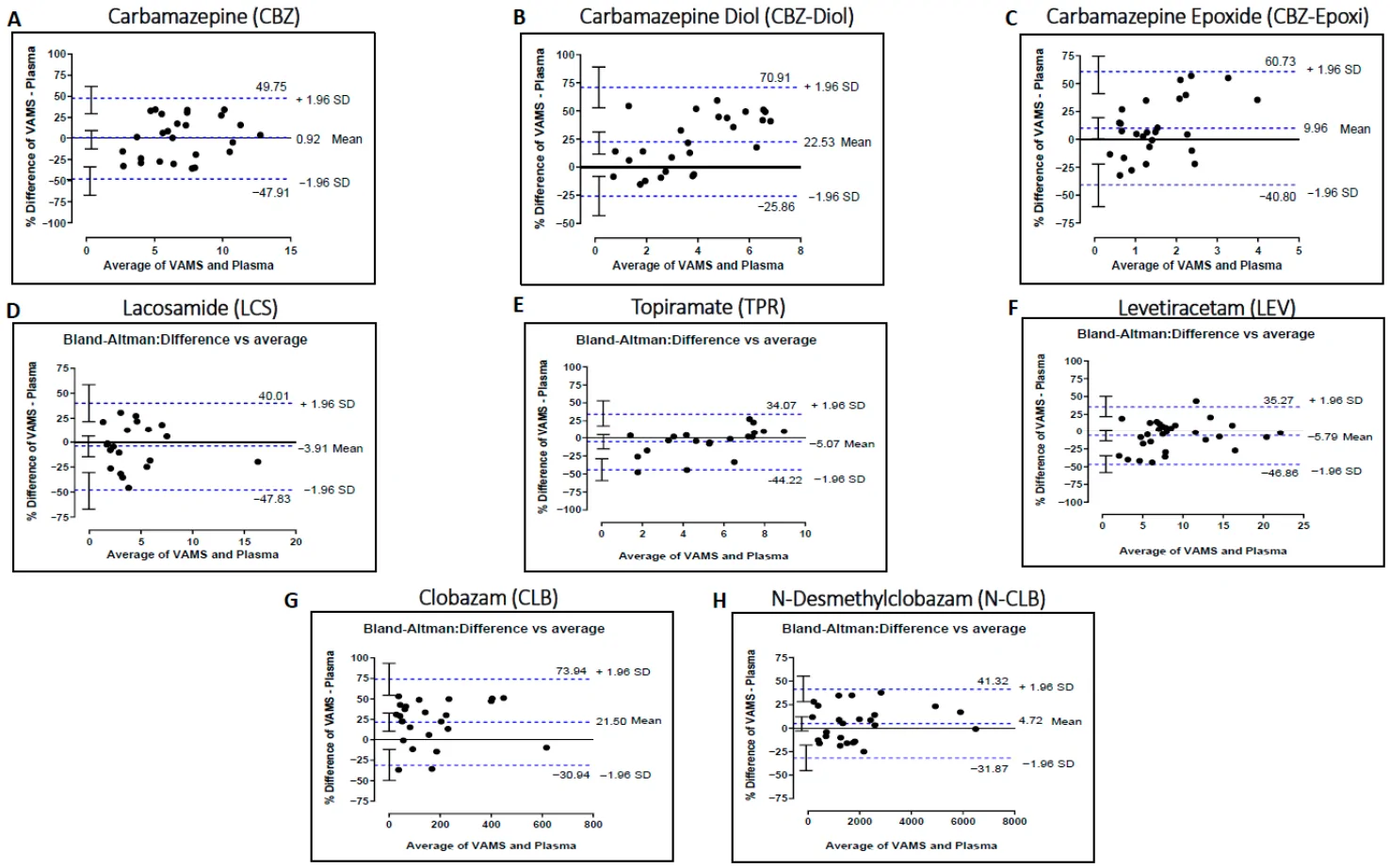
Bland–Altman plots of carbamazepine (**A**), carbamazepine-diol (**B**), carbamazepine-epoxide (**C**), lacosamide (**D**), topiramate (**E**), levetiracetam (**F**), clobazam (**G**), N-Desmethylclobazam (**H**) concentrations measured in plasma and VAMS samples. Dotted horizontal blue lines are drawn at the mean difference (bias) and 95% limits of agreement, which are defined as the mean difference ± 1.96 times the standard deviation of the differences. Continuous vertical black lines indicate the 95% confidence interval (CI) for the mean and the agreement limits.

Bland–Altman plots were used to further compare plasma versus VAMS drug concentrations (Figure 4). In particular, data are displayed as scatter diagrams of the % difference plotted against the average of plasma and VAMS measurements. Dotted horizontal blue lines are drawn at the mean difference (bias) and 95% limits of agreement, which are defined as the mean difference ± 1.96 times the standard deviation of the differences. The % mean difference values are displayed in Figure 4 and also reported with the relative 95% CI in Table 7. Exclusively for CBZ-Diol, -Epoxi and clobazam, the line of equality was not included in the 95% CI of mean difference (Figure 4). Moreover, for these analytes less than 67% of paired samples showed a % difference within ± 20%.

Finally, a Wilcoxon test was used to compare paired drugs’ concentrations measured in both plasma and VAMS samples for each tested analyte, and a significant *p* value (*p* < 0.05) was observed for CBZ-Diol and CLB (Supplementary Table S2).

Considering the poor agreement between VAMS and plasma concentrations for some of the assessed analytes, we explored the possibility of converting VAMS capillary blood to estimated plasma concentrations (EC_pl_). In particular, we have evaluated different experimental approaches based on both blood-to-plasma ratio and the equation regression analysis (i.e., slope and intercept values) [25]. By using Bland–Altman comparison plots, our results demonstrated that conversion based on blood-to-plasma ratio led to a substantial agreement between estimated versus observed plasma concentrations. Conversely, the Passing–Bablok regression analysis still confirmed the presence of a constant and proportional bias for CBZ-Diol, -Epoxi and TPR (Table 8). However, the predictive performance of the conversion approach was also evaluated as median percentage predictive error (MPPE %), revealing a % value within ±15% for all the analyzed compounds. Finally, a Wilcoxon test was used to compare paired observed vs. estimated plasma concentrations, showing no significant differences (Supplementary Table S3). A similar approach was used to estimate plasma concentration from VAMS results by using the hematocrit (HCT%) value for each patient (mean HCT% has been reported in Supplementary Table S3). However, compared to the blood-to-plasma ratio conversion factor, this approach showed a worse performance for all the analytes and led to a poor agreement between estimated versus observed plasma concentrations as assessed by Bland–Altman comparison plots (Supplementary Table S4).

## 3. Discussion

Despite the increasing interest towards DBS as tool for performing TDM of different drug classes, the use of these disposables for quantitative applications is still debated [7]. VAMS has been introduced on the market as an alternative microsampling strategy for quantification of different analytes in small volumes of blood (10–30 µL) collected by fingerprick [10,26]. This aspect is particularly valuable since it allows overcoming the ethical and compliance limitations related to the collection of multiple and large volumes of blood by venipuncture in neonates and children. Moreover, experimental evidence suggests that, compared to DBS, VAMS is not affected by HCT value [6,9]. So far, several published reports have compared concentrations of different drugs measured in VAMS or DBS devices to those found in conventionally collected plasma samples [11,12,13,14,15,16,17,27]. Consequently, VAMS is gradually impacting on the strategy to carry out pharmacokinetic (PK) and toxicokinetic studies [28]. Similarly, VAMS has also been proposed as a viable tool for performing TDM of both old and new-generation ASMs, including cannabidiol [19,20,21,22,23]. Although the above-mentioned studies have validated VAMS with “real-life” clinical samples, in a few of them capillary blood samples were collected for VAMS following patients’ fingerprick [22,23,24].

Here, we have developed and validated two LC-MS/MS methods for contemporary quantification of carbamazepine (CBZ), its metabolites diol (CBZ-Diol) and epoxide (CBZ-Epoxi), levetiracetam (LEV), lacosamide (LCS), topiramate (TPR) and the benzodiazepine (BZ) clobazam (CLB) with its active metabolite N-desmethylclobazam (N-CLB) in VAMS samples. Thereafter, clinical applicability of these methods was tested on “real-life” samples collected from pediatric patients affected by epilepsy. In particular, VAMS samples were collected by a nurse through fingerprick. Subsequently, VAMS concentrations were compared to those found in plasma samples obtained from peripheral venous blood collected through venipuncture. A similar approach has been described in the report published by Cancellerini and colleagues (2024), where the authors compared plasma concentrations of different AEDs with those measured in VAMS samples autonomously collected by patients (self-collected VAMS) and by nurses (nurse-collected VAMS) [24].

However, there are some differences between our study and the previously published report: the first one is the age of patients involved. Here, we have tested the clinical applicability of VAMS in pediatric patients (median age of 4.2 and 6.4 years for males and females, respectively), followed at the Neurology, Epilepsy, and Movement Disorders Unit of our hospital. Recently, in a study published by Cobo-Golpe M. and colleagues (2025), the authors have developed an LC-MS/MS method for eight antiepileptic drugs and two metabolites using DBS and VAMS collected from child and adult patients under treatment with one (*n* = 56) or more (*n* = 24) of the studied AEs [23].

Nevertheless, the ASMs analyzed in our study represent an important novelty compared to the current literature. In fact, although CBZ, CBZ-Epoxi, LEV, TPM and LCS have been already evaluated in previous reports [19,23,24], here we have also assessed the use of VAMS for monitoring blood concentrations of clobazam (CLB) and its active metabolite N-desmethylclobazam (N-CLB). To our knowledge, this is the first study aimed to validate VAMS for measuring blood levels of this benzodiazepine in pediatric patients. Moreover, compared to the study published by Cancellerini et al., here we have newly developed and validated two LC-MS/MS bioanalytical methods: one for the contemporary quantification of CBZ, CBZ-Diol, CBZ-Epoxi, LEV, TPM and LCS and another one for measuring CLB and N-CLB in VAMS samples. In particular, we have used mobile phases, gradient conditions and the chromatographic column of the ClinMass^®^ TDM Kit (Recipe Chemicals^®^) for contemporary determination of ASMs in plasma samples. However, our methods for VAMS have been further validated in accordance with the ICH M10 guidelines for bioanalytical method validation (https://www.ema.europa.eu/en/ich-m10-bioanalytical-method-validation-scientific-guideline. Accessed on 4 October 2022) [29]. Specifically, our results in terms of inter-day and intra-day accuracy and precision for both QC samples and LLOQ were in line with bioanalytical validation guidelines and previously published reports [19,20]. Similarly, our methods were selective and specific due to the absence of interfering peaks in six different VAMS samples prepared from drug-free whole blood samples and spiked with the internal standard (IS). These data agree with the results shown in the study conducted by D’Urso and colleagues in which the authors collected VAMS samples by dipping tips into tubes containing whole blood [19]. Moreover, absence of interfering compounds is an important aspect to consider since epilepsy treatment is often based on the co-administration of different ASMs.

Extraction recovery is an important parameter to consider for an accurate quantification of drug concentrations in VAMS samples. Our results for the analyzed VAMS samples prepared at low and high QC levels were within the acceptable range (94 to 102%). These results, alongside matrix effect evaluation, were in line with the ICH M10 requirements and with previously published studies on ASM quantification in VAMS samples [19,20].

Stability is essential for promoting remote TDM with VAMS. In fact, the opportunity of self-collection at home could be advantageous for managing pediatric patients with epilepsy and for avoiding travel to hospitals or reference laboratories exclusively to monitor drug levels. Freshly collected VAMS samples can be left to dry for at least 3 h and shipped in storage bags to reference laboratories. Here, we have simulated a hypothetical “real-life” situation in which VAMS samples were self-collected at the patient’s home and shipped to a laboratory within three days. In our study, indeed, after collecting VAMS samples by fingerprick, these disposables were left to dry at room temperature for at least three hours. Thereafter, samples were stored in desiccant bags for three days before being analyzed through LC-MS/MS. In contrast, plasma samples were processed and analyzed on the same day of VAMS collection in accordance with the TDM protocol established as clinical practice in our center. Given that our stability data demonstrated a compound- and concentration-dependent degradation over time, particularly at low concentrations, it is plausible that part of the observed discrepancies—especially for less stable analytes such as CBZ-Diol, CBZ-Epoxi and clobazam—may reflect preanalytical degradation during storage rather than true differences between capillary whole blood and plasma matrices. This aspect should be taken into account when evaluating the agreement between methods.

However, we have evaluated VAMS samples’ long-term stability by storing these devices in storage bags kept at room temperature for up to 28 days. Our results showed that stability of VAMS samples was both compound- and concentration-dependent. In fact, the % differences calculated between the initial (Time 0) concentration and that found after 7 days were lower at medium and high QC levels for all the evaluated compounds. CBZ-Epoxi and LEV also showed a lower % difference compared to CBZ-Diol, LCS and TPR at a low QC concentration. For CBZ, % differences after 7 days were higher compared to other compounds at low, medium and high QC levels. In our hands, the % differences after 14 and 28 days showed a time-dependent increase suggesting compromised stability of VAMS samples. Therefore, based on our results and a previously published report [19], an interval of 7–10 days represents the maximum time frame to guarantee reliable results for samples shipped to a reference laboratory. However, our storage conditions were different from those previously described [19]. Similarly, our results for CBZ-Epoxi were in line with those previously reported at 7 days but not after 1 month [20]. On the other hand, stability results observed for CBZ did not agree with data shown by Velghe and Stove [20]. A possible explanation for this discrepancy could be related to the different concentrations of prepared QC samples. In fact, in the study of Velghe and Stove [20], low, medium and high QC concentrations were higher than those selected in our study. This could explain the higher stability observed for up to 1 month [20].

In our study, for the first time, stability of clobazam and its active metabolite N-desmethylclobazam in VAMS samples was evaluated. Similar to the other compounds, stability of CLB and N-CLB at 7, 14 and 28 days was higher when evaluating medium and high QCs, although a time-dependent increase in % differences was observed. In contrast, CLB and N-CLB VAMS samples prepared at low QC showed higher % difference compared to medium and high concentrations, especially after 14 and 28 days for N-CLB and after 14 days for CLB.

Stability evaluated after two freeze–thaw cycles in prepared QC samples showed low % difference compared to the initial concentrations for all the analyzed compounds. This aspect suggests that samples could be frozen at −20 °C after preparation and analyzed with reliably for up to 1 month in case of temporary unavailability of LC-MS/MS instruments.

According to the recent guidelines published by the International Association of Therapeutic Drug Monitoring and Clinical Toxicology [25], we have firstly evaluated the agreement between VAMS and plasma for drug concentrations by using Passing–Bablok regression analysis and Bland–Altman plots. Based on our results, a constant bias was found for CBZ-Diol, -Epoxi and TPR since the 95% CI intercept did not include 0. For the same analytes, a proportional bias was also observed since the 95% CI slopes did not include 1. Moreover, a Wilcoxon test used to compare paired plasma and VAMS concentrations showed a significant *p* value for CBZ-Diol (*p* < 0.001) and CLB (*p* < 0.01). For Bland–Altman analysis, the acceptance criteria were that the mean difference between the two measurements should be <10% with no individual value > 20% [30]. Commonly, about 70–80% of the samples must lie within a 20% difference. In our hands, for CBZ-Diol, CBZ-Epoxi and CLB, only 46%, 54% and 40%, respectively, of paired samples were within the acceptance criteria. However, it is also worth mentioning that part of the discrepancy may be attributable not only to matrix differences but also to preanalytical factors such as compound stability and storage-related degradation of VAMS samples.

Considering the poor agreement observed for some analytes, we have evaluated the potential of converting VAMS capillary blood results to estimated plasma concentrations (EC_pl_) [25]. In fact, plasma represents the standard matrix for TDM routine analysis, and discrepancies between capillary blood and plasma concentration could compromise a clinician’s interpretation of VAMS results. Moreover, therapeutic ranges are often established through plasma-based assays. Therefore, converting capillary whole blood results to plasma concentrations could improve the validity of microsampling procedures and facilitate the interpretation of TDM results. Specifically, we have determined a conversion factor using different experimental approaches based on both blood-to-plasma ratio and the equation of Passing–Bablok regression analysis. In particular, consistency of blood-to-plasma ratio (R) for each compound is crucial for the accuracy of VAMS measurements. In our study, R values were calculated as previously described and were comparable to those reported and validated in the literature [19,24]. However, for CLB and N-CLB we were not able to compare R values due to the lack of reference values. Therefore, we have used the blood-to-plasma R to convert VAMS results into estimated plasma concentrations according to Equation (8) reported by Boffel L. and colleagues [25]. Compared to the Passing–Bablok-derived conversion factors, this approach showed a better performance and led to a substantial agreement between estimated versus observed plasma concentrations as assessed by Bland–Altman comparison plots. Conversely, the Passing–Bablok regression analysis still confirmed the presence of a constant and proportional bias for CBZ-Diol, -Epoxi and TPR. Although topiramate (TPR) exhibited evidence of both proportional and constant bias in Passing–Bablok regression analysis, the overall agreement between VAMS and plasma concentrations was considered clinically acceptable. In particular, Bland–Altman analysis showed that the mean bias and limits of agreement for TPR fell within predefined acceptability criteria, with a substantial proportion of paired samples lying within a ±20% difference. Moreover, the application of conversion approaches did not lead to a meaningful improvement in agreement or clinical interpretability. In contrast, for CBZ-Diol, CBZ-Epoxi and clobazam (CLB), the presence of bias was associated with a poorer agreement and a lower proportion of samples within acceptable limits, thus supporting the use of conversion factors for these analytes. Therefore, the recommendation to apply conversion factors was based on the clinical relevance of the observed differences rather than solely on the statistical identification of bias.

Finally, the Wilcoxon test used to compare paired observed vs. estimated plasma concentrations did not show significant differences. Therefore, based on these results and the recently published guidelines [25], blood-to-plasma ratio could be used to convert capillary blood concentrations measured using VAMS to the estimated plasma levels, allowing clinicians to compare the obtained results with plasma therapeutic ranges. Alternatively, when capillary blood and plasma concentrations are not interchangeable and compound distribution is unknown, conversion factors based on the equation regression analysis could be advisable [25].

Although different research groups have previously reported that the volume of blood sampled with VAMS seems to be independent of the HCT value [6,9], we wanted to explore the effect of this parameter on the estimation of plasma concentration values. In particular, we have used the HCT% value of each patient to convert VAMS results into estimated plasma concentrations according to Equation (4) reported by Boffel L. and colleagues [25]. As depicted in Supplementary Table S4, compared to the blood-to-plasma ratio conversion factor, this approach showed a worse performance for all the analytes and led to a poor agreement between estimated versus observed plasma concentrations as assessed by Bland–Altman comparison plots. Therefore, in terms of conversion factors, our results suggest the use of blood-to-plasma ratios instead of the hematocrit value. However, it is important to underline that, before applying the proposed conversion approaches, an appropriate standardization of the preanalytical conditions (i.e., evaluation of compound stability and samples’ storage) should be considered.

ASMs reported in this study were selected since they are among the most frequently used and routinely monitored in our pediatric clinical practice, representing the majority of TDM requests in our center. Moreover, they encompass different pharmacological classes and heterogeneous pharmacokinetic behaviors (including variability in metabolism, protein binding, and blood–plasma distribution), allowing us to evaluate VAMS performance across clinically relevant and representative scenarios. The number of paired samples analyzed in this study and the obtained results are in line with previous studies in which VAMS samples (collected through patients’ fingerprick or by dipping tips into whole blood tubes) have been clinically tested for TDM of ASMs in a “real-world” setting [19,20,23,24].

We are aware that a limitation of our study is the low number of paired samples collected for some analytes (i.e., TPR and LCS). However, it is worth mentioning that, in our hands, the numbers of analyzed samples were in line with previous studies in which VAMS samples (collected through patients’ fingerprick or by dipping tips into whole blood tubes) have been clinically tested for TDM of ASMs in a “real-world” setting [19,20,23,24].

Another limitation of our study is the absence of paired VAMS and plasma samples (distinct from those collected within the study), to be used as an external and independent data set for further validating the adopted conversion factor. Similarly, we have to acknowledge as an additional limitation of our study the absence of incurred sample reanalysis (ISR) or repeat analysis of authentic patient VAMS samples. ISR is recognized as an important approach to further demonstrate the reproducibility of a bioanalytical method when applied to real clinical specimens, beyond the performance assessed using calibrators and quality control samples. In the present study, however, ISR was not performed due to ethical and regulatory constraints. In fact, in study protocol approved by the local Ethics Committee, the number of VAMS samples collected for each patient was limited to one. Therefore, we were not able to collected duplicate or multiple VAMS samples from the same patient in order to perform a repeated extraction and reanalysis. Although this represents a limitation, it is worth noting that the analytical method was thoroughly validated according to ICH M10 guidelines and applied to a large cohort of real-life pediatric patients, partially supporting the robustness of the obtained results. Future studies specifically designed to include ISR on capillary VAMS samples are warranted to further strengthen confidence in the reproducibility of this approach in routine therapeutic drug monitoring.

Conversely, advantages of our study are represented by the ex novo development and validation of two bioanalytical methods for the contemporary quantification of four AEs and one BZ in a single run by using the same chromatographic column and mobile phases. Moreover, this is a “real-life” study in which VAMS samples have been collected by fingerprick in a cohort of pediatric patients and, thereafter, tested for TDM of ASMs during the routine clinical practice. Finally, this is the first study aimed to validate VAMS for monitoring blood levels of clobazam and its active metabolite in pediatric patients receiving ASMs.

Due to the low blood volume required, VAMS is a compliant alternative to conventional venipuncture for pediatric patients and represents a valid opportunity for promoting remote TDM in the future. In fact, the stability of dried samples and the easy logistics allow shipment of VAMS samples without need for refrigeration. This aspect could be particularly valuable for pediatric patients who are unable sometimes to reach specialized hospitals, due to their pathological condition or to the long travel distance. However, in order to limit preanalytical biases that could affect drug measurements in home self-samples, an adequate training on VAMS collection, storage, and shipment should be given to nurses or patients’ caregivers. In fact, this aspect guarantees an appropriate reproducibility across different laboratories.

Finally, in order to improve the use of these disposables, national healthcare systems should implement evidence-based policy interventions to facilitate adoption and ensure adequate distribution of microsampling devices within routine clinical practice.

## 4. Materials and Methods

### 4.1. Chemical Reagents and Equipment

Drug-free whole blood samples were collected in EDTA tubes from healthy volunteers at the Blood Transfusion Center of the Bambino Gesù Children’s Hospital, IRCCS (Rome, Italy) after giving informed consent. Whole blood samples were used for preparation of calibrators (CALs), low-, medium- and high-quality control samples (QCs), and blank matrices used in selectivity and specificity assessments. After collection, samples were aliquoted and stored at room temperature (RT) until use.

Volumetric absorptive microsampling (VAMS) devices (Mitra^®^ tips, 10 µL) were sourced from Neoteryx (Torrance, CA, USA) and maintained at room temperature (approximately + 20 °C) until use. The specific lot of VAMS devices used in this study was accompanied by a certificate of conformance, indicating an average blood absorption volume of 10.34 µL.

### 4.2. Human Samples

VAMS samples were collected from *n* = 121 pediatric patients affected by epilepsy and followed at the Neurology, Epilepsy, and Movement Disorders Unit of Bambino Gesù Children’s Hospital, IRCCS in Rome (Italy). All subjects were receiving antiseizure therapy based on AEs and/or BZ combinations. Detailed demographic data of the study population are provided in Supplementary Table S5.

Peripheral blood samples were drawn into EDTA tubes by conventional venipuncture from each recruited patient. All samples were collected in the morning 30 min before therapy (trough concentration, C*_trough_*) in accordance with the therapeutic drug monitoring (TDM) program established at Bambino Gesù Children’s Hospital during routine clinical practice. At the same time, capillary blood samples were collected through fingerprick on VAMS devices by trained nursing staff following the manufacturer’s instructions.

Plasma samples were recovered from venous blood samples by centrifuging at 3500 rcf for 5 min and subjected to the routine TDM of ASMs using the commercially available ClinMass^®^ TDM Kit (Recipe chemicals^®^, Dessauerstraße 38, 80992 München, Germany). After collection VAMS samples were left to dry for at least two hours at room temperature (RT). Thereafter, samples were stored in a darkened desiccant bag at RT for three days before sample preparation.

Before VAMS collection, informed consent for participation in this comparative study was obtained from the parents or legal representatives of all subjects under 18 years of age. This study was conducted in observation of the ethical principles established by the Declaration of Helsinki. Prior to initiation, the research protocol was evaluated and approved by the Ethics Committee of the Bambino Gesù Children’s Hospital (2430_OPBG_2021).

### 4.3. Determination of Antiepileptic (AE) and Benzodiazepine (BZ) Levels in Plasma and VAMS Samples by LC-MS/MS

The following ASMs were included in the analyses: carbamazepine (CBZ), carbamazepine-diol (CBZ-Diol), carbamazepine-epoxide (CBZ-Epoxi), lacosamide (LCS), topiramate (TPR), levetiracetam (LEV), clobazam (CLB), and norclobazam (N-CLB).

Measurements were performed at the Laboratory of Metabolic Diseases and Drug Biology of Bambino Gesù Children’s Hospital in Rome, Italy. Instrumentation consisted of high-performance liquid chromatography (HPLC) coupled with tandem mass spectrometry (MS/MS). Specifically, an Agilent 1290 Infinity II UHPLC system (Agilent Technologies, Deutschland GmbH, Waldbronn, Germany) was integrated with an Agilent 6470 triple quadrupole mass spectrometer, equipped with an ESI-JET-STREAM ion source operating in positive ionization mode (ESI+). Data acquisition and quantitative analysis of selected analytes were carried out using MassHunter Workstation Software, version 10.1 (Agilent Technologies, Deutschland GmbH, Waldbronn, Germany).

### 4.4. Calibration Standards and Quality Control Samples

The ClinMass^®^ TDM Kit (Recipe Chemicals^®^, Dessauerstraße 38, 80,992 Munich, Germany) was used for quantification of CBZ, CBZ-Diol, CBZ-Epoxi, LCS, TPR, LEV, CLB, and N-CLB in fresh plasma samples. This commercially available kit comprises lyophilized calibrators (CALs) and quality controls (QCs) for each selected compound. According to the manufacturer’s instructions, lyophilized CALs and QCs were reconstituted with 1 mL of purified water (Milli-Q Plus) and stored at −80 °C until analysis. Concentrations of calibrators for the selected AEs and BZs were as follows: CBZ: 1.46, 7.56, 21.3 µg/mL; CBZ-Diol: 0.506, 2.57, 8.03 µg/mL; CBZ-Epoxi: 0.458, 2.51, 7.27 µg/mL; LCS: 0.924, 5.03, 14.8 µg/mL; LEV: 4.19, 21.9, 67.1 µg/mL; TPR: 1.22, 5.95, 17.7 µg/mL; CLB: 29.2, 154, 490 ng/mL; N-CLB: 253, 1391, 4369 ng/mL.

Target concentrations of low and high QCs in plasma were as follow: CBZ: 4.48 and 9.96 µg/mL; CBZ-Diol: 1.68 and 3.83 µg/mL; CBZ-Epoxi: 1.91 and 4.27 µg/mL; LCS: 2.91 and 6.69 µg/mL; LEV: 12.7 and 28.6 µg/mL; TPR: 3.42 and 8.07 µg/mL; CLB: 95.7 and 330 ng/mL; N-CLB: 827 and 2883 ng/mL.

Similarly, six-level calibration curves were prepared for quantification of the selected AEs and BZs in VAMS samples by performing serial dilution of drug-free whole blood samples spiked at different concentrations. Thereafter, tubes were gently agitated at 300 rpm using a ThermoMixer C (Eppendorf s.r.l., Milan, Italy) at room temperature (RT) for 20 min. Calibration curves were prepared to cover the therapeutic ranges described in the literature for each analyte (Consensus Guidelines for Therapeutic Drug Monitoring in Neuropsychopharmacology: Update 2017) [31]. Calibrators used for determination of AEs and BZs in VAMS samples were the following: CBZ: 0.74, 1.48, 3.99, 7.97, 10.75, 21.50 µg/mL; CBZ-Diol: 0.29, 0.57, 1.39, 2.77, 4.44, 8.88 µg/mL; CBZ-Epoxi: 0.29, 0.59, 1.57, 3.14, 4.24, 8.47 µg/mL; LCS: 0.43, 0.85, 2.38, 4.76, 6.90, 13.80 µg/mL; LEV: 1.95, 3.90, 10.80, 21.60, 31.55, 63.10 µg/mL; TPR: 0.60, 1.20, 2.92, 5.83, 9.05, 18.10 µg/mL; CLB: 12.6, 25.2, 69, 138, 206.5, 413 ng/mL; N-CLB: 90, 180, 529.5, 1059, 1575, 3150 ng/mL.

VAMS CALs and QCs were prepared by carefully dipping the lower part of a 10 μL tip into EDTA tubes containing whole blood spiked at nominal concentrations. In particular, after introducing the VAMS device into the tube, the blood surface was touched until the white tip was completely filled by capillarity. A full immersion into tubes was avoided to prevent tip overfilling. Thereafter, VAMS CALs and QCs were left to air dry in a dedicated rack to prevent samples from touching each other for at least 3 h at RT until the analysis.

For VAMS, nominal concentrations of low, medium and high QCs were as follows: CBZ: 1.38, 6.70 and 12.0 µg/mL; CBZ-Diol: 0.89, 3.93 and 5.70 µg/mL; CBZ-Epoxi: 0.65, 3.32 and 6.17 µg/mL; LCS: 0.61, 5.02 and 9.06 µg/mL; LEV: 2.54, 20.83 and 45.0 µg/mL; TPR: 0.90, 5.89 and 11.30 µg/mL; CLB: 31.9, 165.0 and 330.0 ng/mL; N-CLB: 165.4, 1441.5 and 2400 ng/mL.

### 4.5. Sample Preparation and Extraction

Plasma CALs, QCs and patients’ samples were prepared according to the instructions provided by the ClinMass^®^ TDM Kit (Recipe chemicals^®^, Dessauerstraße 38, 80992 München, Germany) for determination of CBZ, CBZ-Diol, CBZ-Epoxi, LCS, TPR, LEV, CLB, and N-CLB levels. Briefly, 50 µL of each calibrator, QC and plasma sample was mixed with 100 µL of Internal Standard Mix provided by the kit in amber Eppendorf tubes. After mixing for 30 s and centrifuging at 13,000 rpm for 9 min at RT, 100 µL of supernatant from each sample was transferred to a vial and injected into the LC-MS/MS system.

For the analysis of VAMS CALs, QCs and patients’ samples, VAMS devices were rehydrated by placing the tips into amber Eppendorf tubes containing 100 µL of purified water (Milli-Q Plus water) and shaken for 10 s. The collection water was mixed with 100 µL of Internal Standard Mix (included in the ClinMass^®^ TDM Kit) and agitated at 600 rpm for 1 h at room temperature on a ThermoMixer C (Eppendorf s.r.l, 20159 Milan, Italy). Thereafter, tubes were centrifuged at 13,000 rpm for 9 min at RT. Finally, 100 µL of supernatant from each sample was transferred to a vial and injected into the LC-MS/MS system.

CALs and QCs for plasma and VAMS samples were freshly prepared before analysis and were run within each analytical sequence. Thereafter, they were discarded after use (within 24 h of preparation).

### 4.6. Bioanalytical Validation

The ClinMass^®^ TDM Kit (Recipe chemicals^®^, Dessauerstraße 38, 80992 München, Germany) used in this study is certified CE/IVD and is already validated according to the ICH M10 guidelines on bioanalytical method validation and study sample analysis; 25 July 2022 EMA/CHMP/ICH/172948/2019, Committee for Medicinal Products for Human Use. (Available at: https://www.ema.europa.eu/en/ich-m10-bioanalytical-method-validation-scientific-guideline. Accessed on 4 October 2022) [29].

However, in order to measure CBZ, CBZ-Diol, CBZ-Epoxi, LEV, TPR, LCS, CLB and N-CLB in VAMS samples, we have developed two bioanalytical methods: one for contemporary detection of AEs and another for BZs by using the mobile phases, gradient conditions and the chromatographic column of the ClinMass^®^ TDM Kit. Thereafter, these methods were validated according to the ICH M10 guidelines on bioanalytical method validation. In particular, we have evaluated accuracy, precision, selectivity, specificity, matrix effect, recovery and presence of carry-over. Moreover, stability of VAMS samples stored at room temperature for up to 28 days was also assessed. For all experiments the acceptance criteria were set for precision (expressed as % coefficient of variation, CV) at ≤15% (≤20% of the LLOQ) and for accuracy (expressed as mean %bias) at ≤15% (≤20% of the LLOQ).

#### 4.6.1. Accuracy and Precision

Intra-day and inter-day accuracy and precision were evaluated across *n* = 10 independent experiments, each including VAMS QC at low, medium and high levels, conducted over a four-month period. Accuracy was expressed as the mean percentage bias, while precision was defined as the percentage coefficient of variation (%CV). Acceptable accuracy was within ± 15%, with a tolerance of ±20% for the LLOQ. Similarly, precision (%CV) was required to remain below 15% for all levels, except for the LLOQ, where a maximum of ±20% was allowed.

#### 4.6.2. Selectivity and Specificity

Selectivity and specificity were assessed by verifying the absence of interfering signals in six VAMS blank samples with and without the internal standard. To confirm method selectivity, the median signal detected in these blank samples was required to remain below 20% of the LLOQ. Blank whole blood samples were collected in EDTA tubes from healthy volunteers at the Blood Transfusion Center of the Children’s Hospital Bambino Gesù, after obtaining informed consent.

#### 4.6.3. Carry-Over

Carry-over was assessed by injecting three VAMS blank samples immediately following the highest calibration standard. In accordance with ICH M10 recommendations, carry-over was considered negligible if the signal detected in the blank samples did not exceed 20% of the LLOQ and 5% of the IS response.

#### 4.6.4. Matrix Effect and Extraction Recovery

Matrix effect (ME) and extraction recovery (ER) were assessed for CBZ, CBZ-Diol, CBZ-Epoxi, LCS, TPR, LEV, CLB, and N-CLB on VAMS devices at low and high QC levels (*n* = 3). Analyses were performed using six distinct pools of blank matrix (neat) obtained from individual healthy donors. ME was calculated as (B/A) × 100%, where B represents the peak area of each analyte spiked into a blank matrix extract (post-extraction spiking), and A corresponds to the peak area of the analyte at the same concentration in a pure solution [32]. ER was determined as (C/B) × 100%, with C being the peak area of each analyte spiked into the blank matrix prior to extraction. Acceptable ranges were defined as 85–115% for ME% and 90–110% for ER%.

#### 4.6.5. Stability

Stability was investigated by analyzing the concentrations of CBZ, CBZ-Diol, CBZ-Epoxi, LCS, TPR, LEV, CLB, and N-CLB in VAMS samples prepared at low, medium and high QC levels and stored in desiccant bags at room temperature for up to 28 days. The percentage difference was calculated between the drugs’ initial concentration (preparation day, Time 0) and after 7, 14 and 28 days.

Additionally, stability was also assessed in extracted VAMS samples prepared at low and high QC levels after two freeze–thaw cycles over 1 month at −20 °C. To evaluate stability, the percentage difference between postthaw and initial concentrations was calculated. According to the ICH M10 guidelines, a deviation within ±15% was deemed acceptable.

### 4.7. Conversion of Capillary Blood Microsampling Results

Plasma concentrations of antiepileptic drugs and benzodiazepines obtained from venous blood samples were used as references. Different strategies to convert VAMS concentrations to plasma concentrations were evaluated by using various experimental approaches as previously described [25]. Among these experimental approaches, the blood-to-plasma ratio (R value) was calculated by dividing concentrations of each AE and BZ obtained in capillary blood samples collected through VAMS with that measured in plasma [19]. The average R value (± SD) for each drug was used to calculate the estimated plasma concentration (EC_pl_) according to Equation (8) described by Boffel L and colleagues (2025) [25]. The obtained R values were comparable with those previously reported [19,24]. Similarly, alternative conversion approaches based on HCT% and both slope and intercept (calculated from Passing–Bablok regression analyses) were also used in accordance with Equation (4) and Equations (11) and (12), respectively [25].

Finally, the predictive performance of each conversion approach was evaluated by calculating the median percentage predictive error (MPPE %) according to Equation (14) [25]. An MPPE % within ± 15% was considered analytically acceptable [33].

### 4.8. Statistical Analysis

Agreement between drug concentrations measured in conventional (fresh) plasma and those obtained with VAMS was evaluated by using non-parametric Passing–Bablok regression [34] and the Spearman correlation coefficient. The 95% confidence intervals (CIs) for both slope and intercept were also calculated. To further assess the comparability between sampling methods (fresh plasma vs. VAMS), Bland–Altman analysis was performed by plotting the differences against the mean concentrations of ASMs measured in plasma and VAMS samples [35]. The mean difference (bias) and standard deviation (SD) of the differences were also computed. The acceptance criterion for Bland–Altman plots was that the mean difference between the measurements should be lower than 10% with no individual value > 20% [30]. Comparison between paired plasma and VAMS drug concentrations was also evaluated with the non-parametric Wilcoxon test. *p* < 0.05 was considered significant. All statistical analyses were conducted using GraphPad Prism software version 9 (GraphPad Software, San Diego, CA, USA).

## 5. Conclusions

The use of VAMS devices for monitoring ASM blood levels represents a significant step forward in making TDM more accessible, ethical, and sustainable. This microsampling method is particularly advantageous in pediatric settings, where the reduced blood volume required and the possibility of performing self-sampling at home significantly improve patient compliance and overall well-being. It is worth mentioning that, before their introduction into clinical practice, VAMS samples need to be validated by comparing measured blood concentrations with those obtained in plasma collected by traditional sampling approaches (i.e., venous vs. capillary blood).

In this study, we have developed and validated two LC-MS/MS bioanalytical methods in accordance with ICH M10 guidelines for bioanalytical method validation. Our results confirm previously published studies on the clinical utility of VAMS for performing TDM of ASMs during routine clinical practice. Moreover, the use of these microsampling devices for TDM of clobazam and its active metabolite in a cohort of pediatric patients has been evaluated for the first time in this study. Therefore, our study highlights the utility of VAMS for TDM of selected ASMs in pediatric patients during routine clinical practice.

## Figures and Tables

**Table 1:** Summary of key data and main findings of this study.

Item	Details
**Population**	Real-life observational comparative study conducted on 121 pediatric patients (median age ~4–6 years) during routine TDM practice
**Sampling Strategy**	VAMS collected via fingerprick and plasma samples obtained through conventional venipuncture
**Analytes**	CBZ, CBZ-Diol, CBZ-Epoxi, LEV, LCS, TPR, CLB and N-CLB
**Analytical Approach**	UHPLC–MS/MS methods developed for simultaneous quantification on VAMS and plasma samples
**Method Validation**	Validated according to ICH M10 guidelines (https://www.ema.europa.eu/en/ich-m10-bioanalytical-method-validation-scientific-guideline, accessed on 28 August 2023)
**Comparison Results**	Good agreement for CBZ, LCS, TPR, LEV, and N-CLB; variability for CBZ metabolites and CLB
**Conversion Strategy Proposed**	Blood-to-plasma ratio used to estimate plasma concentrations with acceptable predictive performance (MPPE within ±15%)
**Novelty**	First validation of VAMS for CLB and N-CLB monitoring in pediatric patients
**Clinical Relevance**	Supports feasibility of VAMS for real-life TDM in pediatric epilepsy patients
**VAMS Advantages**	Minimally invasive fingerprick approach suitable for pediatric patients Reduced discomfort compared to venipuncture Very small blood volume required (10–30 µL) Easy storage and shipment without refrigeration, enabling remote TDM Limited hematocrit effect compared to DBS Facilitates routine and potential home-based TDM

**Table 2:** Intra-assay precision (%CV) and accuracy (%bias) for LLOQ and QCs of Carbamazepine (CBZ), -Diol (CBZ-Diol), -Epoxide (CBZ-Epoxi), Lacosamide (LCS), Levetiracetam (LEV), Topiramate (TPR), Clobazam (CLB) and its active metabolite N-Desmethylclobazam (N-CLB).

**CBZ**	**Parameter**				
	**Quality control sample (target concentration)**	**LLOQ (0.75 µg/mL)**	**L-QC (1.38 µg/mL)**	**M-QC (6.7 µg/mL)**	**H-QC (12.0 µg/mL)**
	**Number of analyzed samples**	10	10	10	10
	**Carbamazepine concentration found µg/mL (median, range)**	0.83 (0.70–0.91)	1.32 (1.30–1.33)	7.08 (7.03–7.11)	10.37 (10.34–10.41)
	**Intra-assay accuracy (%bias)**	11.11	−4.59	5.72	−13.58
	**Intra-assay precision (%CV)**	13.91	1.16	0.65	0.35
**CBZ-DIOL**	**Quality control sample (target concentration)**	**LLOQ (0.32 µg/mL)**	**L-QC (0.89 µg/mL)**	**M-QC (3.93 µg/mL)**	**H-QC (5.7 µg/mL)**
	**Number of analyzed samples**	10	10	10	10
	**Carbamazepine-diol concentration found µg/mL (median, range)**	0.30 (0.25–0.35)	0.77 (0.72–0.80)	4.38 (4.37–4.41)	6.23 (5.34–7.02)
	**Intra-assay accuracy (%bias)**	−6.25	−13.86	11.54	9.36
	**Intra-assay precision (%CV)**	16.67	5.43	0.53	13.56
**CBZ-EPOXI**	**Quality control sample (target concentration)**	**LLOQ (0.27 µg/mL)**	**L-QC (0.65 µg/mL)**	**M-QC (3.32 µg/mL)**	**H-QC (6.17 µg/mL)**
	**Number of analyzed samples**	10	10	10	10
	**Carbamazepine-epoxide concentration found µg/mL (median, range)**	0.31 (0.27–0.35)	0.59 (0.56–0.60)	3.41 (3.40–4.00)	5.55 (5.00–6.31)
	**Intra-assay accuracy (%bias)**	13.58	−9.74	2.81	−10.10
	**Intra-assay precision (%CV)**	13.18	3.94	0.45	12.28
**LCS**	**Parameter**				
	**Quality control sample (target concentration)**	**LLOQ (0.35 µg/mL)**	**L-QC (0.61 µg/mL)**	**M-QC (5.02 µg/mL)**	**H-QC (9.06 µg/mL)**
	**Number of analyzed samples**	10	10	10	10
	**Lacosamide concentration found µg/mL (median, range)**	0.36 (0.30–0.40)	0.63 (0.55–0.70)	4.89 (4.86–4.92)	9.73 (9.72–9.74)
	**Intra-assay accuracy (%bias)**	1.90	3.83	−2.59	7.43
	**Intra-assay precision (%CV)**	14.39	12.06	0.61	0.12
**LEV**	**Quality control sample (target concentration)**	**LLOQ (1.95 µg/mL)**	**L-QC (2.54 µg/mL)**	**M-QC (20.83 µg/mL)**	**H-QC (45.0 µg/mL)**
	**Number of analyzed samples**	10	10	10	10
	**Levetiracetam concentration found µg/mL (median, range)**	2.00 (1.69–2.30)	2.90 (2.85–2.94)	23.57 (23.45–23.67)	37.80 (34.97–40.03)
	**Intra-assay accuracy (%bias)**	2.39	14.04	13.14	−13.99
	**Intra-assay precision (%CV)**	15.28	1.56	0.47	6.84
**TPR**	**Quality control sample (target concentration)**	**LLOQ (0.60 µg/mL)**	**L-QC (0.90 µg/mL)**	**M-QC (5.89 µg/mL)**	**H-QC (11.30 µg/mL)**
	**Number of analyzed samples**	10	10	10	10
	**Topiramate concentration found µg/mL (median, range)**	0.65 (0.62–0.75)	1.00 (0.90–1.10)	5.19 (4.98–5.71)	10.06 (10.02–10.58)
	**Intra-assay accuracy (%bias)**	12.22	11.11	−10.13	−9.56
	**Intra-assay precision (%CV)**	10.11	10.00	7.10	3.06
**CLB**	**Parameter**				
	**Quality control sample (target concentration)**	**LLOQ (12.6 ng/mL)**	**L-QC (31.9 ng/mL)**	**M-QC (165.0 ng/mL)**	**H-QC (330.0 ng/mL)**
	**Number of analyzed samples**	10	10	10	10
	**Clobazam concentration found ng/mL (median, range)**	11.60 (11.0–12.40)	32.96 (32.0–34.74)	183.0 (173.0–193.0)	340.33 (312.0–383.0)
	**Intra-assay accuracy (%bias)**	−7.94	3.31	10.91	3.13
	**Intra-assay precision (%CV)**	6.22	4.69	7.73	11.05
**N-CLB**	**Quality control sample (target concentration)**	**LLOQ (90.0 ng/mL)**	**L-QC (165.4 ng/mL)**	**M-QC (1441.5 ng/mL)**	**H-QC (2400 ng/mL)**
	**Number of analyzed samples**	10	10	10	10
	**N-desmetilclobazam concentration found ng/mL (median, range)**	101.27 (94.90–104.92)	182.62 (155.17–198.41)	1297.94 (1153.83–1400.0)	2501.0 (2485.0–2517.0)
	**Intra-assay accuracy (%bias)**	12.53	10.41	−9.96	4.21
	**Intra-assay precision (%CV)**	5.47	13.07	9.89	0.64

**Table 3:** Inter-assay precision (%CV) and accuracy (%bias) for LLOQ and QCs of Carbamazepine (CBZ), -Diol (CBZ-Diol), -Epoxide (CBZ-Epoxi), Lacosamide (LCS), Levetiracetam (LEV), Topiramate (TPR), Clobazam (CLB) and its active metabolite N-Desmethylclobazam (N-CLB).

**CBZ**	**Parameter**				
	**Quality control sample (target concentration)**	**LLOQ (0.75 µg/mL)**	**L-QC (1.38 µg/mL)**	**M-QC (6.7 µg/mL)**	**H-QC (12.0 µg/mL)**
	**Number of analyzed samples**	**10**	**10**	**10**	**10**
	**Carbamazepine concentration found µg/mL (median, range)**	**0.86 (0.58–0.98)**	**1.31 (1.28–1.34)**	**6.39 (5.64–7.33)**	**11.0 (9.34–11.96)**
	**Inter-assay accuracy (%bias)**	**14.67**	**−4.83**	**−4.63**	**−8.33**
	**Inter-assay precision (%CV)**	**18.70**	**2.33**	**11.27**	**10.55**
**CBZ-DIOL**	**Quality control sample (target concentration)**	**LLOQ (0.32 µg/mL)**	**L-QC (0.89 µg/mL)**	**M-QC (3.93 µg/mL)**	**H-QC (5.7 µg/mL)**
	**Number of analyzed samples**	10	10	10	10
	**Carbamazepine-diol concentration found µg/mL (median, range)**	0.34 (0.30–0.38)	0.77 (0.73–0.80)	4.10 (3.92–4.39)	6.35 (5.94–6.59)
	**Inter-assay accuracy (%bias)**	6.25	−13.48	4.41	11.32
	**Inter-assays precision (%CV)**	16.00	4.68	6.13	4.81
**CBZ-EPOXI**	**Quality control sample (target concentration)**	**LLOQ (0.27 µg/mL)**	**L-QC (0.65 µg/mL)**	**M-QC (3.32 µg/mL)**	**H-QC (6.17 µg/mL)**
	**Number of analyzed samples**	10	10	10	10
	**Carbamazepine-epoxide concentration found µg/mL (median, range)**	0.29 (0.20–0.35)	0.57 (0.51–0.62)	3.17 (2.57–3.40)	5.33 (4.36–6.00)
	**Inter-assay accuracy (%bias)**	6.67	−12.92	−4.58	−13.55
	**Inter-assay precision (%CV)**	19.86	7.76	11.0	13.42
**LCS**	**Parameter**				
	**Quality control sample (target concentration)**	**LLOQ (0.35 µg/mL)**	**L-QC (0.61 µg/mL)**	**M-QC (5.02 µg/mL)**	**H-QC (9.06 µg/mL)**
	**Number of analyzed samples**	10	10	10	10
	**Lacosamide concentration found µg/mL (median, range)**	0.35 (0.31–0.43)	0.61 (0.55–0.77)	5.14 (4.52–6.05)	8.67 (7.61–9.70)
	**Inter-assay accuracy (%bias)**	1.14	0.33	2.31	−4.33
	**Inter-assays precision (%CV)**	14.21	14.77	12.39	9.34
**LEV**	**Quality control sample (target concentration)**	**LLOQ (1.95 µg/mL)**	**L-QC (2.54 µg/mL)**	**M-QC (20.83 µg/mL)**	**H-QC (45.0 µg/mL)**
	**Number of analyzed samples**	10	10	10	10
	**Levetiracetam concentration found µg/mL (median, range)**	2.14 (1.80–2.60)	2.88 (2.50–3.10)	22.51 (19.22–24.49)	38.42 (35.62–40.0)
	**Inter-assay accuracy (%bias)**	9.74	13.19	8.06	−14.61
	**Inter-assay precision (%CV)**	13.86	9.15	8.84	4.32
**TPR**	**Quality control sample (target concentration)**	**LLOQ (0.60 µg/mL)**	**L-QC (0.90 µg/mL)**	**M-QC (5.89 µg/mL)**	**H-QC (11.30 µg/mL)**
	**Number of analyzed samples**	10	10	10	10
	**Topiramate concentration found µg/mL (median, range)**	0.56 (0.50–0.68)	1.00 (0.80–1.15)	6.36 (5.51–7.94)	12.15 (9.0–12.59)
	**Inter-assays accuracy (%bias)**	−4.58	10.00	11.10	1.50
	**Inter-assay precision (%CV)**	14.78	14.46	13.52	14.50
**CLB**	**Parameter**				
	**Quality control sample (target concentration)**	**LLOQ (12.6 ng/mL)**	**L-QC (31.9 ng/mL)**	**M-QC (165.0 ng/mL)**	**H-QC (330.0 ng/mL)**
	**Number of analyzed samples**	10	10	10	10
	**Clobazam concentration found ng/mL (median, range)**	11.7 (11.4–12.0)	33.13 (31.52–34.74)	146.73 (132.59–160.86)	326.13 (280.78–358.15)
	**Inter-assay accuracy (%bias)**	−7.14	3.86	−11.08	−1.17
	**Inter-assay precision (%CV)**	2.56	4.86	9.63	12.38
**N-CLB**	**Quality control sample (target concentration)**	**LLOQ (90.0 ng/mL)**	**L-QC (165.4 ng/mL)**	**M-QC (1441.5 ng/mL)**	**H-QC (2400 ng/mL)**
	**Number of analyzed samples**	10	10	10	10
	**N-desmetilclobazam concentration found ng/mL (median, range)**	108.27 (94.90–125.0)	176.21 (166.0–186.3)	1269.97 (1170.0–1370.0)	2360.5 (2260.7–2460.0)
	**Inter-assay accuracy (%bias)**	19.6	6.53	−11.90	−1.65
	**Inter-assay precision (%CV)**	14.16	5.76	7.87	4.22

**Table 4:** Results of matrix effect (ME) and extraction recovery (ER) experiments for Carbamazepine (CBZ), -Diol (CBZ-Diol), -Epoxide (CBZ-Epoxi), Lacosamide (LCS), Levetiracetam (LEV), Topiramate (TPR), Clobazam (CLB) and its active metabolite N-Desmethylclobazam (N-CLB) prepared at Low and High QC levels (*n* = 3).

**CBZ**		**L-QC (1.38 µg/mL)**		**H-QC (12.0 µg/mL)**	
		**ER%**	**ME%**	**ER%**	**ME%**
		102.0 ± 4.6	112.0 ± 10.5	99.3 ± 2.83	110.0 ± 8.0
	**Number of samples**	3	3	3	3
**CBZ-DIOL**		**L-QC (0.89 µg/mL)**		**H-QC (5.7 µg/mL)**	
		**ER%**	**ME%**	**ER%**	**ME%**
		101.6 ± 4.5	94.6 ± 1.9	97.3 ± 3.2	95.6 ± 3.0
	**Number of samples**	3	3	3	3
**CBZ-EPOXI**		**L-QC (0.65 µg/mL)**		**H-QC (6.17 µg/mL)**	
		**ER%**	**ME%**	**ER%**	**ME%**
		98.0 ± 3.0	111.0 ± 12.0	96.0 ± 4.5	112.0 ± 11.5
	**Number of samples**	3	3	3	3
**LCS**		**L-QC (0.61 µg/mL)**		**H-QC (9.06 µg/mL)**	
		**ER%**	**ME%**	**ER%**	**ME%**
		98.0 ± 4.8	95.0 ± 3.6	94.6 ± 3.5	95.3 ± 4.0
	**Number of samples**	3	3	3	3
**LEV**		**L-QC (2.54 µg/mL)**		**H-QC (45.0 µg/mL)**	
		**ER%**	**ME%**	**ER%**	**ME%**
		97.6 ± 1.1	98.3 ± 1.9	94.0 ± 1.8	98.6 ± 3.9
	**Number of samples**	3	3	3	3
**TPR**		**L-QC (0.90 µg/mL)**		**H-QC (11.30 µg/mL)**	
		**ER%**	**ME%**	**ER%**	**ME%**
		99.7 ± 3.9	98.6 ± 2.4	97.0 ± 3.1	98.1 ± 4.4
	**Number of samples**	3	3	3	3
**CLB**		**L-QC (31.9 ng/mL)**		**H-QC (330.0 ng/mL)**	
		**ER%**	**ME%**	**ER%**	**ME%**
		97.0 ± 3.6	86.6 ± 2.3	94.3 ± 2.5	94.6 ± 3.4
	**Number of samples**	3	3	3	3
**N-CLB**		**L-QC (165.4 ng/mL)**		**H-QC (2400 ng/mL)**	
		**ER%**	**ME%**	**ER%**	**ME%**
		101.0 ± 4.0	103.0 ± 1.21	95.0 ± 1.9	105.0 ± 5.0
	**Number of samples**	3	3	3	3

**Table 5:** Stability data for Carbamazepine (CBZ), -Diol (CBZ-Diol), -Epoxide (CBZ-Epoxi), Lacosamide (LCS), Levetiracetam (LEV), Topiramate (TPR), Clobazam (CLB) and N-Desmethylclobazam (N-CLB) at Low, Medium and High QC levels (*n* = 3). Data are presented as the percentage difference between the concentration measured at Time 0 (VAMS preparation) and following 7, 14 and 28 days of storage at room temperature (RT).

Analyte	VAMS Stability (% Difference)			
	QC Samples	Time Point		
		Day 7	Day 14	Day 28
**CBZ**	**Low (1.38 µg/mL)**	58.02	60.7	62.5
	**Medium (6.7 µg/mL)**	52.2	58.7	65.8
	**High (12.0 µg/mL)**	39.5	42.4	62.9
**CBZ-DIOL**	**Low (0.89 µg/mL)**	84.1	93.4	96.1
	**Medium (3.93 µg/mL)**	8.15	19.1	34.9
	**High (5.7 µg/mL)**	1.2	33.8	34.1
**CBZ-EPOXI**	**Low (0.65 µg/mL)**	5.82	12.0	28.0
	**Medium (3.32 µg/mL)**	6.19	7.59	19.38
	**High (6.17 µg/mL)**	2.69	9.87	29.57
**LCS**	**Low (0.61 µg/mL)**	29.00	45.90	60.87
	**Medium (5.02 µg/mL)**	6.66	19.35	38.36
	**High (9.06 µg/mL)**	8.80	17.22	33.10
**LEV**	**Low (2.54 µg/mL)**	2.70	14.43	32.40
	**Medium (20.83 µg/mL)**	0.14	5.44	20.08
	**High (45.0 µg/mL)**	8.66	14.84	19.25
**TPR**	**Low (0.90 µg/mL)**	29.54	31.60	34.37
	**Medium (5.89 µg/mL)**	3.43	4.21	19.34
	**High (11.30 µg/mL)**	12.19	18.44	20.93
**CLB**	**Low (31.9 ng/mL)**	16.98	32.54	46.53
	**Medium (165.0 ng/mL)**	8.91	14.46	20.82
	**High (330.0 ng/mL)**	8.94	10.16	42.44
**N-CLB**	**Low (165.4 ng/mL)**	8.62	85.60	93.08
	**Medium (1441.5 ng/mL)**	14.75	20.20	40.08
	**High (2400 ng/mL)**	21.02	31.31	40.13

**Table 6:** Freeze–thaw stability data for Carbamazepine (CBZ), -Diol (CBZ-Diol), -Epoxide (CBZ-Epoxi), Lacosamide (LCS), Levetiracetam (LEV), Topiramate (TPR), Clobazam (CLB) and its active metabolite N-Desmethylclobazam (N-CLB) prepared at Low and High QC levels (*n* = 3). Data are presented as the percentage difference between the concentration measured at Time 0 (sample preparation) and after two freeze–thaw cycles over 1 month.

Analyte	Freeze–Thaw Stability (% Difference)		
	QC Samples	−20 °C Freeze–Thaw Cycle	
		First	Second
**CBZ**	**Low (1.38 µg/mL)**	1.82	0.1
	**High (12.0 µg/mL)**	0.75	0.76
**CBZ-DIOL**	**Low (0.89 µg/mL)**	1.18	6.13
	**High (5.7 µg/mL)**	0.54	1.6
**CBZ-EPOXI**	**Low (0.65 µg/mL)**	2.22	1.12
	**High (6.17 µg/mL)**	1.43	0.94
**LCS**	**Low (0.61 µg/mL)**	1.18	2.73
	**High (9.06 µg/mL)**	1.20	1.70
**LEV**	**Low (2.54 µg/mL)**	0.51	1.45
	**High (45.0 µg/mL)**	0.27	0.04
**TPR**	**Low (0.90 µg/mL)**	0.83	11.08
	**High (11.30 µg/mL)**	9.28	12.18
**CLB**	**Low (31.9 ng/mL)**	1.78	3.61
	**High (330.0 ng/mL)**	3.30	6.71
**N-CLB**	**Low (165.4 ng/mL)**	3.96	2.00
	**High (2400 ng/mL)**	1.87	0.94

**Table 7:** Comparison between sampling methods: whole blood collected through VAMS vs. Plasma collected by venipuncture.

	Passing–Bablok					Bland–Altman		Spearman	
	Slope	95% CI	Intercept	95% CI	R^2^	Bias	95% CI	rho	95% CI
**CBZ** **(*n* = 26)**	0.8725	0.6588 to 1.086	0.6900	−0.6326 to 2.013	0.67	0.92	−9.141–10.99	0.83	0.64 to 0.92
**CBZ-Diol** **(*n* = 26)**	0.5592	0.4703 to 0.6481	0.7898	0.3554 to 1.224	0.85	22.53	12.55–32.50	0.93	0.85 to 0.97
**CBZ-Epoxi (*n* = 26)**	0.6232	0.4560 to 0.7905	0.3654	0.1662 to 0.5645	0.76	9.96	−0.496–20.43	0.92	0.83 to 0.96
**LEV** **(*n* = 30)**	1.003	0.8598 to 1.145	0.2828	−0.7588 to 1.324	0.88	−5.79	−13.62–2.03	0.91	0.82 to 0.96
**TPR** **(*n* = 20)**	0.8206	0.6924 to 0.9488	0.9518	0.3175 to 1.586	0.86	−5.07	−14.42–4.28	0.91	0.78 to 0.96
**LCS** **(*n* = 20)**	1.166	0.6281 to 1.704	−0.4969	−2.418 to 1.424	0.91	−3.91	−14.40–6.57	0.93	0.83 to 0.97
**CLB** **(*n* = 25)**	0.7905	0.3647 to 1.216	0.3306	−53.74 to 54.40	0.83	21.50	10.45–32.54	0.95	0.88 to 0.98
**N-CLB** **(*n* = 25)**	0.8807	0.7103 to 1.051	98.35	−152.6 to 349.3	0.95	4.72	−2.98–12.43	0.95	0.88 to 0.98

**Table 8:** Comparison between Estimated vs. Observed plasma concentrations.

	Passing–Bablok					Bland–Altman		Spearman	
	Slope	95% CI	Intercept	95% CI	R^2^	Bias	95% CI	rho	95% CI
**CBZ** **(*n* = 26)**	0.9150	0.6910 to 1.139	0.6378	−0.6952 to 1.971	0.67	−2.93	−12.99–7.135	0.83	0.64 to 0.92
**CBZ-Diol** **(*n* = 26)**	0.7405	0.6220 to 0.8591	0.7434	0.3136 to 1.173	0.85	−3.16	−13.25–6.916	0.93	0.85 to 0.97
**CBZ-Epoxi (*n* = 26)**	0.7286	0.5288 to 0.9284	0.3479	0.1447 to 0.5512	0.76	−3.84	−14.39–6.716	0.92	0.83 to 0.96
**LEV** **(*n* = 30)**	0.9602	0.8228 to 1.098	0.3053	−0.7341 to 1.345	0.88	−1.76	−9.59–6.06	0.91	0.82 to 0.96
**TPR** **(*n* = 20)**	0.7939	0.6696 to 0.9182	0.9628	0.3249 to 1.601	0.86	−2.07	−11.44–7.294	0.91	0.78 to 0.96
**LCS** **(*n* = 20)**	1.142	0.6124 to 1.671	−0.4923	−2.420 to 1.435	0.91	−1.91	−12.39–8.58	0.93	0.83 to 0.97
**CLB** **(*n* = 25)**	1.045	0.4678 to 1.621	−3.270	−61.28 to 54.74	0.83	−3.44	−14.48–7.292	0.95	0.88 to 0.98
**N-CLB** **(*n* = 25)**	0.9439	0.7613 to 1.127	95.39	−156.2 to 347.0	0.95	−1.98	−9.70–5.731	0.95	0.88 to 0.98

## Data Availability

The original contributions presented in this study are included in the article/Supplementary Material. Further inquiries can be directed to the corresponding author.

## Supplementary Materials

The following supporting information can be downloaded at: https://www.mdpi.com/article/10.3390/ph19081188/s1, Figure S1: Six-point calibration curves used for measuring drug concentrations in VAMS samples. Table S1: Calibration ranges for the selected antiseizure medications. Table S2: Comparison between unconverted whole blood VAMS vs. Plasma drugs’ concentrations. Table S3: Comparison between Observed vs. Estimated Plasma drug concentrations by using blood-to-plasma ratio. Table S4: Comparison between Estimated vs. Observed plasma concentrations by using the HCT% value. Table S5: Demographic characteristics of patients.
