# Supplementary material for: Volumetric Absorptive Microsampling (VAMS) for Therapeutic Drug Monitoring of Antiseizure Medications (ASMs) in Pediatric Patients
Source: Pharmaceuticals (Basel). 2026 Jul 29;19(8):1188. doi: 10.3390/ph19081188 (PMC13516248; doi:10.3390/ph19081188)
Supplement: Supplementary file 1 [file pharmaceuticals-19-01188-s001.zip › Supplementary Table S1.pdf]

Supplementary Table S1. Calibration ranges for the selected antiseizure medications.

	CBZ	CBZ-DIOL	CBZ-EPOXI	LCS	TPR	LEV	CLB	N-CLB
**Calibration range**	0.74 – 21.50	0.29 – 8.88	0.29 - 8.47	0.43 - 13.80	0.60 - 18.10	1.95 – 63.10	12.6 – 413	90.0 – 3150
**Calibration curve equation**	Y=0.33*X – 0.10	Y=0.003*X – 4.09E-004	Y=0.07*X – 0.004	Y = 0.01*X + 2.0E-004	Y = 0.04*X + 0.01	Y = 0.01*X – 0.01	Y = 4.65E-005*X – 2.13E-004	Y = 7.9E-006*X + 8.57E-004
**Regression coefficient (R^2)**	0.99	0.99	0.99	0.99	0.99	0.98	0.99	0.99
