# Supplementary material for: Volumetric Absorptive Microsampling (VAMS) for Therapeutic Drug Monitoring of Antiseizure Medications (ASMs) in Pediatric Patients
Source: Pharmaceuticals (Basel). 2026 Jul 29;19(8):1188. doi: 10.3390/ph19081188 (PMC13516248; doi:10.3390/ph19081188)
Supplement: Supplementary file 1 [file pharmaceuticals-19-01188-s001.zip › Supplementary Table S2.pdf]

Supplementary Table S2. Comparison between unconverted whole blood VAMS vs Plasma drugs' concentrations.

	CBZ (µg/mL)	CBZ-DIOL (µg/mL)	CBZ-EPOXI (µg/mL)	LCS (µg/mL)	TPR (µg/mL)	LEV (µg/mL)	CLB (ng/mL)	N-CLB (ng/mL)
**Number of correlations (n)**	26	26	26	20	20	30	25	25
**Therapeutic range#**	4 -12	n.a.	0.12 - 8.1##	1 - 10	2 - 8	10 - 40	30 – 300	300 – 3000
**Observed Plasma mean concentration ± SD (range min-max)**	6.82 ± 2.68 (2.87 - 12.5)	3.23 ± 1.46 (0.73 – 5.73)	1.41 ± 0.73 (0.40 – 3.28)	4.50 ± 3.62 (1.19 – 17.90)	5.22 ± 2.16 (1.39 – 8.52)	9.15 ± 5.09 (2.19 – 22.30)	148 ± 140 (24.2 – 646)	1847 ± 1567 (161 – 6523)
**VAMS mean concentration ± SD (range min-max)**	7.03 ± 2.99 (2.27-13.0)	4.37 ± 2.50 (0.68 – 8.25)	1.68 ± 1.11 (0.35-4.69)	4.29 ± 3.13 (1.47-14.7)	5.20 ± 2.60 (1.35 – 9.40)	8.85 ± 5.08 (1.71 – 21.90)	187 ± 174 (30.4 ± 587)	1986 ± 1774 (181 – 6462)
**p-value**	0.532	p < 0.001	0.101	0.522	0.869	0.280	p < 0.01	0.275

#Consensus Guidelines for Therapeutic Drug Monitoring in Neuropsychopharmacology: Update 2017. Pharmacopsychiatry. 2018 Jan;51(1-02):9-62. doi: 10.1055/s-0043-116492.

##Volumetric Absorptive Microsampling: A New Sampling Tool for Therapeutic Drug Monitoring of Antiepileptic Drugs. Ther. Drug Monit. 2019, 41, 681–692.
