# Supplementary material for: Volumetric Absorptive Microsampling (VAMS) for Therapeutic Drug Monitoring of Antiseizure Medications (ASMs) in Pediatric Patients
Source: Pharmaceuticals (Basel). 2026 Jul 29;19(8):1188. doi: 10.3390/ph19081188 (PMC13516248; doi:10.3390/ph19081188)
Supplement: Supplementary file 1 [file pharmaceuticals-19-01188-s001.zip › Supplementary Table S3.pdf]

Supplementary Table S3. Comparison between Observed vs Estimated Plasma drug concentrations by using blood-to-plasma ratio.

	CBZ (μg/mL)	CBZ-DIOL (μg/mL)	CBZ-EPOXI (μg/mL)	LCS (μg/mL)	TPR (μg/mL)	LEV (μg/mL)	CLB (ng/mL)	N-CLB (ng/mL)
**Number of correlations (n)**	26	26	26	20	20	30	25	25
**Therapeutic range**[#]	4 -12	n.a.	0.12 - 8.1[##]	1 - 10	2 - 8	10 - 40	30 – 300	300 – 3000
**Observed mean concentration ± SD (range min-max)**	6.82 ± 2.68 (2.87 - 12.5)	3.23 ± 1.46 (0.73 – 5.73)	1.41 ± 0.73 (0.40 – 3.28)	4.50 ± 3.62 (1.19 – 17.9)	5.22 ± 2.16 (1.39 – 8.52)	9.15 ± 5.09 (2.19 – 22.3)	148 ± 140 (24.2 – 646)	1847 ± 1567 (161 – 6523)
**Estimated mean concentration ± SD (range min-max)**	6.76 ± 2.88 (2.18-12.5)	3.36 ± 1.93 (0.52 – 6.35)	1.46 ± 0.97 (0.30-4.08)	4.38 ± 3.19 (1.50-15.0)	5.36 ± 2.68 (1.39 – 9.69)	9.22 ± 5.29 (1.78 – 22.9)	154 ± 135 (23.6 ± 455)	1856 ± 1658 (169 – 6039)
**p-value**	0.909	0.439	0.706	0.812	0.475	0.903	0.979	0.676
**Mean HCT% (range min-max)**	0.40 (0.34 - 0.45)	0.40 (0.34 - 0.45)	0.40 (0.34 - 0.45)	0.40 (0.36 – 0.44)	0.39 (0.33 – 0.44)	0.40 (0.30 – 0.46)	0.39 (0.33 – 0.45)	0.39 (0.33 – 0.45)
**Blood-to-plasma ratio R (mean ± SD)**	1.04 ± 0.26	1.30 ± 0.32	1.15 ± 0.31	0.98 ± 0.22	0.97 ± 0.18	0.96 ± 0.20	1.29 ± 0.32	1.07 ± 0.20

[#]Consensus Guidelines for Therapeutic Drug Monitoring in Neuropsychopharmacology: Update 2017. Pharmacopsychiatry. 2018 Jan;51(1-02):9-62. doi: 10.1055/s-0043-116492.

[##]Volumetric Absorptive Microsampling: A New Sampling Tool for Therapeutic Drug Monitoring of Antiepileptic Drugs. Ther. Drug Monit. 2019, 41, 681–692.
