# Supplementary material for: Volumetric Absorptive Microsampling (VAMS) for Therapeutic Drug Monitoring of Antiseizure Medications (ASMs) in Pediatric Patients
Source: Pharmaceuticals (Basel). 2026 Jul 29;19(8):1188. doi: 10.3390/ph19081188 (PMC13516248; doi:10.3390/ph19081188)
Supplement: Supplementary file 1 [file pharmaceuticals-19-01188-s001.zip › Supplementary Table S4.pdf]

Supplementary Table S4. Comparison between Estimated vs Observed plasma concentrations by using the HCT% value.

	Passing-Bablok					Bland-Altman		Spearman	
	**Slope**	**95 % CI**	**Intercept**	**95 % CI**	**R^2**	**Bias**	**95 % CI**	**rho**	**95 % CI**
**CBZ (n=26)**	0.6772	0.4335 to 0.9210	1.535	-0.4477 to 3.518	0.63	12.31	1.78 - 22.83	0.71	0.44 to 0.86
**CBZ-Diol (n=26)**	0.3910	0.2626 to 0.5194	0.9072	0.2693 to 1.545	0.62	46.48	31.21 - 61.75	0.82	0.62 to 0.92
**CBZ-Epoxi (n=26)**	0.4974	0.2799 to 0.7149	0.2991	-0.067 to 0.6659	0.66	39.03	22.25 - 55.81	0.88	0.75 to 0.95
**LEV (n=30)**	0.8529	0.7372 to 0.9686	0.9268	-0.3751 to 2.229	0.93	6.46	-1.80 - 14.73	0.92	0.84 to 0.96
**TPR (n=20)**	0.8202	0.6982 to 0.9423	0.6577	-0.1603 to 1.476	0.79	4.04	-5.45 - 13.53	0.84	0.66 to 0.93
**LCS (n=20)**	1.148	0.5464 to 1.749	-1.614	-4.374 to 1.145	0.88	23.04	10.95 - 35.14	0.88	0.72 to 0.95
**CLB (n=25)**	0.5637	-0.009 to 1.137	8.315	-65.98 to 82.61	0.68	41.13	22.49 - 59.76	0.78	0.54 to 0.90
**N-CLB (n=25)**	0.6254	0.4119 to 0.8389	154.6	-195.5 to 504.7	0.93	26.78	11.69 - 41.86	0.88	0.75 to 0.95
