# Supplementary material for: Volumetric Absorptive Microsampling (VAMS) for Therapeutic Drug Monitoring of Antiseizure Medications (ASMs) in Pediatric Patients
Source: Pharmaceuticals (Basel). 2026 Jul 29;19(8):1188. doi: 10.3390/ph19081188 (PMC13516248; doi:10.3390/ph19081188)
Supplement: Supplementary file 1 [file pharmaceuticals-19-01188-s001.zip › Supplementary Table S5.pdf]

Supplementary Table S5. Demographic characteristics of patients.

Population, n	121
**Male, n (%)**	60 (49.6)
**Female, n (%)**	61 (50.4)
**Age (years)**	
Male, median (IQR)	4.2 (7.3)
Female, median (IQR)	6.4 (6.0)
**Levetiracetam, n (%)**	30 (24.8)
**Diagnosis:**	
Focal Epilepsy, n (%)	18 (60.0)
Generalized epilepsy, n (%)	6 (20.0)
DEE, n (%)	6 (20.0)
**Dosing regimens:**	
200 mg tid, n (%)	1 (3.3)
250 mg bid, n (%)	2 (6.6)
1000 mg bid, n (%)	1 (3.3)
200 mg bid, n (%)	1 (3.3)
500 mg bid, n (%)	5 (16.8)
300 mg bid, n (%)	4 (13.3)
600 mg bid, n (%)	1 (3.3)
150 mg bid, n (%)	1 (3.3)
Alternative dosing regimens, n (%)	14 (46.8)
**Co-medications:**	
Carbamazepine, n (%)	4 (13.3)
Clobazam, n (%)	2 (6.6)
Topiramate, n (%)	1 (3.3)
Carbamazepine + clobazam, n (%)	1 (3.3)
Topiramate + clobazam, n (%)	1 (3.3)
Topiramate + carbamazepine, n (%)	1 (3.3)
**Carbamazepine (CBZ), CBZ-Diol, CBZ-Epoxi, n (%)**	26 (30.6)
**Diagnosis:**	
DEE, n (%)	4 (16.7)
Focal epilepsy, n (%)	21 (81.2)
Epilepsy with febrile and afebrile seizure, n (%)	1 (2.1)
**Dosing regimens:**	
200 mg bid, n (%)	3 (11.6)
400 mg bid, n (%)	3 (11.6)
300 mg bid, n (%)	2 (7.7)
100 mg + 150 mg/die, n (%)	1 (3.8)
200 mg + 300 mg/die, n (%)	2 (7.7)
80 mg bid, n (%)	1 (3.8)
Alternative dosing regimens, n (%)	14 (53.8)
**Co-medications:**	
Clobazam, n (%)	6 (23.1)
Levetiracetam, n (%)	4 (15.4)
Levetiracetam + clobazam, n (%)	2 (7.7)
Levetiracetam + topiramate, n (%)	1 (3.8)
**Clobazam (CLB), N-Desmethylclobazam, n (%)**	25 (20.6)
**Diagnosis:**	
DEE, n (%)	13 (50.0)
Focal epilepsy, n (%)	10 (44.2)
Epilepsy with febrile and afebrile seizures, n (%)	1 (2.9)
Combined focal and generalized epilepsy, n (%)	1 (2.9)
**Dosing regimens:**	
5 mg bid, n (%)	6 (24.0)
10 mg bid, n (%)	3 (12.0)

2.5 mg bid, n (%)	1 (4.0)
10 mg die, n (%)	1 (4.0)
5 mg die, n (%)	2 (8.0)
5 mg tid, n (%)	1 (4.0)
2.5 mg bid, n (%)	1 (4.0)
Alternative dosing regimens, n (%)	10 (40.0)
**Co-medications:**	
Carbamazepine, n (%)	6 (24.0)
Levetiracetam, n (%)	2 (8.0)
Carbamazepine + levetiracetam, n (%)	2 (8.0)
Topiramate, n (%)	3 (12.0)
Lacosamide + topiramate, n (%)	1 (4.0)
Levetiracetam + topiramate, n (%)	1 (4.0)
Lacosamide, n (%)	4 (16.0)
**Lacosamide, n (%)**	20 (16.5)
**Diagnosis:**	
Focal epilepsy, n (%)	19 (93.4)
DEE, n (%)	1 (6.6)
**Dosing regimens:**	
75 mg bid, n (%)	4 (20.0)
50 mg bid, n (%)	8 (40.0)
50 mg + 75 mg/die, n (%)	4 (20.0)
50 mg bid, n (%)	1 (5.0)
25 mg + 50 mg/die, n (%)	1 (5.0)
25 mg bid, n (%)	1 (5.0)
50 mg + 100 mg/die, n (%)	1 (5.0)
**Co-medications:**	
Topiramate + clobazam, n (%)	1 (5.0)
Topiramate, n (%)	1 (5.0)
Clobazam, n (%)	4 (20.0)
**Topiramate, n (%)**	20 (16.5)
**Diagnosis:**	
DEE, n (%)	8 (40.0)
Focal epilepsy, n (%)	12 (60.0)
**Dosing regimens:**	
50 mg bid, n (%)	6 (30.0)
25 mg + 37.5 mg/die, n (%)	4 (20.0)
50 mg bid, n (%)	2 (10.0)
37.5 mg bid, n (%)	2 (10.0)
25 mg tid, n (%)	2 (10.0)
50 mg + 75 mg/die, n (%)	2 (10.0)
25 mg + 50 mg/die, n (%)	2 (10.0)
**Co-medications:**	
Clobazam, n (%)	3 (15.0)
Levetiracetam, n (%)	1 (5.0)
Lacosamide, n (%)	1 (5.0)
Lacosamide + clobazam, n (%)	1 (5.0)
Levetiracetam + clobazam, n (%)	1 (5.0)

DEE: Developmental and epileptic encephalopathy; bid: *bis in die*; tid: *ter in die.* Other dosing regimens include:

for LEV: 1500 mg x2/die, 750 mgx2/die, 250 mg + 250 mg + 500 mg/die, 400 mg x2/die, 200 mg + 250 mg + 200 mg/die, 150 mg x3/die, 400 mg x 2 /die, 240 mg x2/die, 200 mg + 300 mg/die, 300 mg + 350 mg/die, 200 mg x 2/die, 500 mg + 750 mg/die, 300 mg + 200 mg + 300 mg /die, 350 mg x2/die, 700 mg x2/die, 400 mg + 500 mg /die, 150 mg + 200 mg /die, 750 mg x2/die;

for CBZ: 400 mg x2/die, 80 mg x 2/die, 400 mg x3/die, 60 mg x3/die, 600 mg x2/die, 140 mg x3/die, 100 mg x2/die, 120 mg x 2/die, 400 mg + 200 mg +400 mg/die, 800 mg x2/die, 75 mg x2/die, 150 mg x3/die, 200 mg x 3/die, 220 mg x2/die, 250 mg x 2/die, 200 + 400 mg/die, 200 mg + 600 mg/die, 280 mg + 300 mg/die, 280 mg x2/die, 200 mg/die, 160 mg + 160 mg + 200 mg/die, 250 mg + 350 mg/die, 300 mg +400 mg/die, 200 mg +300 mg/die, 240 mg x2/die, 160 mg x2/die, 120 mg x3/die;

for CLB: 10 mg x2/die, 2.5mg + 5 mg/die, 5 mg + 2.5mg + 5 mg/die, 3mg x 2/die, 2.5 mg/die, 5 mg + 10 mg/die, 10 mg/die, 10 mg + 5 mg/die, 10 mg x3/die, 5 mg x2/die, 5 mg + 2 mg + 5 mg /die, 6 mg + 10 mg/die.
